# Golgi condensation causes intestinal lipid accumulation through HIF-1α-mediated GM130 ubiquitination by NEDD4

**DOI:** 10.1038/s12276-025-01396-2

**Published:** 2025-02-03

**Authors:** Hyunsoo Kim, Channy Park, Xiaofan Wei, Arun Chhetri, Laxman Manandhar, Gyuho Jang, Jaetaek Hwang, Batchingis Chinbold, Chagtsalmaa Chuluunbaatar, Hyug Moo Kwon, Raekil Park

**Affiliations:** 1 Department of Biomedical Science and Engineering, Gwangju Institute of Science and Technology, Gwangju, Republic of Korea; 2 School of Life Sciences, Ulsan National Institute of Science and Technology, Ulsan, Republic of Korea

**Keywords:** Golgi, Obesity

## Abstract

The breakdown of Golgi proteins disrupts lipid trafficking, leading to lipid accumulation in the small intestine. However, the causal mechanism of the effects of Golgi protein degradation on the Golgi structure related to lipid trafficking in the small intestine remains unknown. Here we find that Golgi protein degradation occurs under hypoxic conditions in high-fat-diet-fed mice. Hypoxia-induced degradation promotes structural changes in the Golgi apparatus, termed ‘Golgi condensation’. In addition, hypoxia-inducible factor 1α (HIF-1α) activation enhances Golgi condensation through the ubiquitination and degradation of Golgi matrix protein 130 (GM130), which is facilitated by neural precursor cell expressed developmentally downregulated protein 4 (NEDD4). Golgi condensation upon exposure to hypoxia promotes lipid accumulation, apolipoprotein A1 retention and decreased chylomicron secretion in the intestinal epithelium. Golgi condensation and lipid accumulation induced by GM130 depletion are reversed by exogenous GM130 induction in the intestinal epithelium. Inhibition of either HIF-1α or NEDD4 protects against GM130 degradation and, thereby, rescues cells from Golgi condensation, which further increases apolipoprotein A1 secretion and lipid accumulation both in vivo and in vitro. Furthermore, the HIF-1α inhibitor PX-478 prevents Golgi condensation, which decreases lipid accumulation and promotes high-density lipoprotein secretion in high-fat-diet-fed mice. Overall, our results suggest that Golgi condensation plays a key role in lipid trafficking in the small intestine through the HIF-1α- and NEDD4-mediated degradation of GM130, and these findings highlight the possibility that the prevention of structural modifications in the Golgi apparatus can ameliorate intestinal lipid accumulation in obese individuals.

## Golgi condensation promotes lipid accumulation in obese intestine

This study explores how low oxygen levels affect the Golgi apparatus, a cell structure involved in processing and packaging proteins and lipids. The study used mice and cell models to investigate these effects. They found that HIF-1α causes the Golgi apparatus to condense or shrink, which disrupts its function. This change is linked to increased lipid accumulation in the small intestine. The researchers discovered that HIF-1α plays a crucial role in this process by promoting the degradation of another protein, GM130, which is essential for maintaining Golgi structure, and NEDD4, a E3 ligase, which contributes on GM130 degradation. The findings suggest that preventing Golgi condensation by inhibiting HIF-1α could reduce lipid buildup in the obese intestine. This research provides new insights into how hypoxia affects lipid metabolism and could lead to potential treatments for obesity-related conditions.

This summary was initially drafted using artificial intelligence, then revised and fact-checked by the author.

## Introduction

Lipid trafficking in the small intestine primarily involves lipid production and packaging for delivery as lipoproteins to other tissues^1^. Disruption of lipid trafficking, such as lipoprotein secretion, results in abnormal lipid accumulation within epithelial cells^1^. The Golgi apparatus serves as a crucial suborganelle for lipoprotein maturation, a process necessary for lipoprotein secretion from small intestinal epithelial cells. In addition, the disruption of lipoprotein maturation leads to an increase in lipid accumulation within the epithelial cells of *Grasp55*^−/−^mice^2^.

The Golgi apparatus has a diverse array of functions^3^. Its structural organization can be categorized into three distinct forms: flattened, scattered and condensed^4^. The condensed configuration involves the shrinking and compaction of the Golgi structure from its flattened state. These transformational changes are regulated by various factors, including the presence of GOLPH3 and phosphatidylinositol(4)phosphate (PtdIns(4)P), the degradation of Golgi structural proteins, Golgi stress and cell cycle regulation^5^.

Hypoxia is caused by diminished blood flow^6^ or hypoventilation syndrome^7^ in the body. It can affect the Golgi apparatus through various mechanisms, such as triggering cell death^8^ and degrading Golgi proteins^9^ through impeded protein trafficking from the endoplasmic reticulum (ER) to the Golgi. Hypoxia-inducible factor 1 alpha (HIF-1α), a modulator of the response to hypoxic conditions, notably impacts Golgi function^10^. Hypoxia is a state of stress closely related to lipid metabolism and transport in the small intestine. This connection is relevant to various tissues, including adipose tissue and the small intestine, where hypoxia can occur due to obesity. A concrete example of this finding is the elevation of HIF-1α expression in the adipose tissues of both obese mice and humans^11,12^. Furthermore, the HIF-2α protein is reported to be upregulated in the jejunum of patients with obesity^13^. In particular, HIF-1α activation contributes to increased lipid accumulation and promotes adipose tissue whitening^6^. Although several reports indicate that lipid trafficking and accumulation are affected by hypoxia in obesity models, the morphological changes in the Golgi apparatus during lipid trafficking under hypoxic conditions remain unclear.

Here, we explore the structural changes in the Golgi apparatus under hypoxic conditions and proposed a potential mechanism contributing to lipid accumulation in the small intestine during HFD feeding. HIF-1α induced GM130 degradation to promote Golgi condensation, which further resulted in lipid accumulation in in vivo and in vitro models. NEDD4, an E3 ligase, degraded GM130 through ubiquitination under hypoxic conditions. In addition, a series of mechanisms related to Golgi condensation and lipid accumulation were attenuated through the inhibition of HIF-1α activation. These findings underscore the importance of understanding the intricate relationships among Golgi morphology, cellular responses to hypoxia and lipid metabolism.

## Materials and methods

### Animal models

C57BL/6J mice were purchased from Damul Science (Daejeon, Korea) and housed in a temperature-controlled facility (22 ± 1 °C) on a 12 h light–dark cycle with free access to food and water. All animal protocols and procedures were approved by the Institutional Animal Care and Use Committee of the Gwangju Institute of Science and Technology (Gwangju, Korea). Eight-week-old mice were fed a 10% of kcal fat normal chow diet (NCD) containing 10% of kcal from fat (D12450J, Research Diet) or high-fat diet (HFD) containing 60% of kcal from fat (D12491, Research Diet) for 16 weeks to establish the HFD-fed model. PX-478 (10 mg/kg) was administered by oral gavage three times a week starting at 6 weeks of HFD feeding. Before being sacrificed, the mice were fasted for 16 h. After sacrifice, tissues and blood were collected from the mice. Serum was extracted from the blood via centrifugation (1,000*g* for 10 min). The concentrations of high-density lipoprotein (HDL), low-density lipoprotein (LDL) and triglyceride (TG) were measured at the Laboratory Animal Resource Center (LARC, Gwangju Institute of Science and Technology, Korea). The concentrations of ApoA1, ApoB and ApoB48 in the extracted small intestine, Transwell system medium and serum were measured using an ApoA1 enzyme-linked immunosorbent assay (ELISA) Kit (ab238260, Abcam), an ApoB ELISA Kit (ab190806, Abcam) and an ApoB48 ELISA Kit (MBS028193, MYBioSource), respectively.

### Cell culture and in vitro experiments

RPE-1 and Fhs74Int cells (ATCC) were cultured in Dulbecco’s modified Eagle medium and Hybri-care medium (Gibco), respectively, supplemented with 10% fetal bovine serum, penicillin and streptomycin (Invitrogen). The cells were grown at 37 °C in a humidified 5% CO_2_ atmosphere. Caco-2 cell culture and differentiation were performed according to previous protocols^14^. The Caco-2 cells were cultured in minimum essential medium (MEM) containing 20% fetal bovine serum, 1× MEM nonessential amino acid solution, 100 μg/ml streptomycin, 100 U/ml penicillin and 1× GlutaMax (35050061, Thermo Fisher Scientific). The Transwell system was constructed with 0.4 μm pore size Transwell polycarbonate membranes (CLS3413, Merck, Darmstadt, Germany). The Caco-2 cells were maintained for 3 weeks in Transwells until tight junctions were produced. Tight junctions were verified by measuring transepithelial electrical resistance. The cells were incubated in a hypoxia chamber (cell culture laboratory workstation Invivo2) with 1% O_2_ and 5% CO_2_ to establish hypoxia. In addition to the hypoxia chamber, the cells were treated with either cobalt chloride II (CoCl_2_) or dimethyloxalylglycine (DMOG) to mimic hypoxia. For the conjugation of oleic acid with BSA buffer to induce fatty acid absorption by cells, sodium oleate (O7501, Sigma-Aldrich) was mixed with 24% fatty acid-free BSA (A8806, Sigma-Aldrich) diluted with saline solution and incubated at 37 °C. Intracellular free fatty acid uptake was measured using a free fatty acid uptake kit (ab176768, Abcam).

### Transfection

The p(HA)HIF1alpha (P402A, P564A) and p(HA)HIF1alpha (401delta603)R27G plasmids were gifts from Eric Huang^15^, and the HA-HIF1alpha-pcDNA3 plasmid was obtained from William Kaelin^16^. HA-Nedd4 and HA-Nedd4-C744E were gifts from Allan Weissman^17^. Myc_Golga2 was designed and obtained from VectorBuilder. Short interfering RNAs (siRNAs) (Supplementary Fig. 5a) were mixed with RNAiMAX (13778-150, Invitrogen) in Opti-MEM (Gibco). The cells were treated with a mixture of the plasmid and Lipofectamine 3000 transfection reagent (L3000015, Thermo Fisher) in serum-free media to increase the transfection efficiency and were subsequently incubated. After 4 h, the cells were cultured with 20% serum-containing media for 20 h.

### RNA isolation and real-time qRT‒PCR

The cells were washed with ice-cold 1× PBS and then RNA was extracted with TRIzol (15596018, Ambion) according to the manufacturer’s protocols. Complementary DNA was synthesized from total RNA samples using a Transcriptor First Strand cDNA Synthesis Kit (04897030001, Roche) according to the manufacturer’s protocols. Total cDNA samples were used as templates to perform quantitative Reverse Transcription-Polymerase Chain Reaction (qRT‒PCR) using primers (Supplementary Fig. 5b) and FastStart DNA Master SYBR Green (06402712001, Roche).

### Immunofluorescence staining

The cells were cultured in 12-well plates with 12 mm coverslips (0111520, Marienfeld). At the end of the experiment, the cells were fixed with 4% paraformaldehyde for 20 min at room temperature (RT) and rinsed with 1× PBS three times. The cells were permeabilized with 0.5% Triton X-100 for 5 min at RT and rinsed with 1× PBS three times and blocked with 3% BSA diluted in PBS for 1 h. The primary antibodies (Supplementary Fig. 5c) were diluted in 3% BSA for an overnight reaction at 4 °C, and the cells were rinsed (with 1× PBS) and incubated with secondary antibodies (Supplementary Fig. 5c) for 1 h at RT. After being rinsed with 1× PBS and stained with 4,6-diamidino-2-phenylindole (DAPI, 1:1,000; D1306, Invitrogen) diluted in 3% BSA for 5 min, the fluorescence of the cells grown on coverslips that were mounted on microscope slides was detected with a fluorescence microscope (IX73, Olympus), and the results were analyzed with Olympus cellSens Standard software. The intensity of immunofluorescence staining was measured using ImageJ. The intensity of colocalization was subsequently measured by calculating Mender’s coefficient using the Just Another Colocalization Plugin in ImageJ software. The length of the Golgi cisternae was subsequently measured by manual tracing using ImageJ software. The longest cisternae of the Golgi apparatus, located near the nucleus, were calculated using the software.

### TEM

For transmission electron microscopy (TEM) sample preparation, the mice were perfused with normal saline and then fixed with 2% paraformaldehyde and 2.5% glutaraldehyde in 0.1 M sodium cacodylate buffer, pH 7.4 (15960-01-1L, Electron Microscopy Sciences). After fixation, the small intestine was washed with saline and cut into 1 mm pieces. The tissue samples were placed in fixation buffer, stored at 4 °C and transferred for TEM processing by OBEN (Suwon, Korea).

### RNA-seq and proteomic analyses

The RNA sequencing (RNA-seq) data provided by the Colin R. Goding group^18^ were obtained from Gene Expression Omnibus. The raw data were analyzed using Galaxy. The RNASTAR and DESEQ2 methods were used for alignment and normalization, respectively. The proteomic data were obtained from the Hiroki Kawashima group^19^. The gene set enrichment analysis program was used after the protein ID was converted to the gene ID.

### Nuclear fractionation

A nuclear extraction kit (ab113474, Abcam) was used for nuclear and cytoplasmic fractionation. The cells (3 × 10^6^) were collected for nuclear extraction. The extracted nuclear and cytoplasmic proteins were collected and subjected to western blotting.

### Protein preparation and western blotting

The cells were washed and collected in ice-cold PBS supplemented with protease and phosphatase inhibitors to prevent HIF-1α degradation. The cells were lysed in RIPA buffer. The protein concentration was subsequently measured with a Pierce BCA protein assay kit (23225, Thermo Scientific). The tissue was washed with ice-cold PBS and placed in prefilled tube kits/triple-Pure High Impact Ziroconium Beads 3.0 mm (D1032-30, Benchmark Scientific) with RIPA buffer. The tissues were homogenized with a BeadBug6 bead homogenizer (D1036-E, Benchmark Scientific). The protein concentration was measured with a Bio-Rad protein assay kit (I5000001, Bio-Rad). A PageRuler Prestained Protein Ladder (26616 and 26619, Thermo Scientific) was used as the protein ladder. Equivalent amounts of each protein extract were loaded in each well, separated by sodium dodecyl phosphate-polyacrylamide gel electrophoresis, transferred to a nitrocellulose membrane, washed with 1× TBS-T and blocked with 5% skim milk mixed with 1× TBS-T at RT for 1 h. The membrane was incubated with a primary antibody (Supplementary Fig. 5c) diluted in 3% BSA at 4 °C overnight. The membrane was washed three times with 1× TBS-T for 5 min and then incubated with a secondary antibody (Supplementary Fig. 5c) diluted in 3% skim milk at RT for 1 h. After the membrane was washed with 1× TBS-T three times for 5 min, it was placed in an enhanced chemiluminescence solution (34580, Thermo Scientific) and then visualized using a ChemiDoc Touch Imaging System (Bio-Rad).

### IP assay

A Pierce Co-Immunoprecipitation Kit (26149, Thermo Scientific) was used for the immunoprecipitation (IP) assay. The assay was performed according to the manufacturer’s protocol. After IP, the samples were subjected to western blotting and visualized using a ChemiDoc system.

### ChIP assay

The chromatin IP (ChIP) assay was performed with a ChIP assay kit (17-295, Sigma‒Aldrich) according to the manufacturer’s protocol. For the shearing process, the cells were sheared with a Cole Parmer high-intensity ultrasonic sonicator (EW-04714-51, Cole-Palmer) (four sets of 10 s pulses). Every process was performed on ice to reduce DNA damage.

### Oil Red O staining

The tissues were fixed with 4% formaldehyde overnight. The samples were rinsed with 1× PBS, incubated with 10% sucrose for 3 h, rinsed again with 1× PBS and transferred to 30% sucrose for an incubation at 4 °C overnight. After sucrose fixation, the tissues were placed in Cryomold Tissue Tek (4566, Sakura) with Optical Cutting Temperature (OCT) compound (4583, Sakura). Cryosectioning was performed, and Oil red O staining was performed according to the manufacturer’s protocol^20^.

### Lipid extraction and GC‒MS analysis

The lipids were extracted from cells according to the Bligh and Dyer method^21^. The extracted lipids were diluted in 200 μl of hexane before the gas chromatography and mass spectrometry (GC‒MS) analysis. The gas chromatography parameters were as follows: injection temperature 230 °C, pressure 49.7 kPa, total flow rate 24.0 ml/min, column flow rate 1.00 ml/min, linear velocity 36.1 cm/s and purge flow 3.0 ml/min. Mass spectrometry was performed with an ion source temperature of 200 °C and an interface temperature of 300 °C.

### Statistical analysis/graphical tool

All the statistical comparisons were analyzed via a Student’s *t*-test to determine whether the two sets of data were significantly different from each other. The differences with *P* values <0.05 were considered statistically significant. All graphs of the data were drawn with Prism 9.

## Results

### HFD results in lipid accumulation and hypoxia in the small intestine

**Fig. 1:**
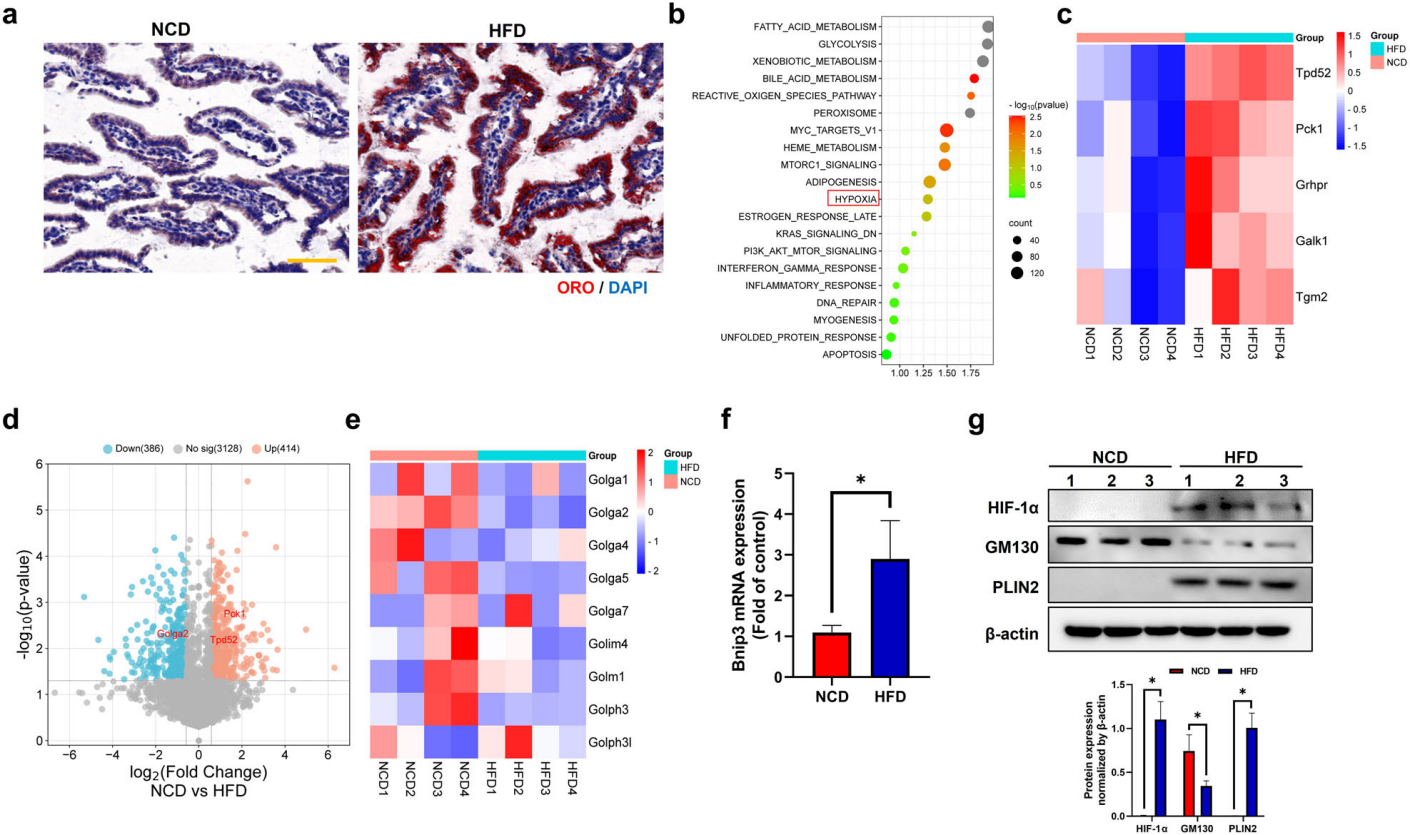
Lipid accumulation and increased expression of hypoxia-related proteins are observed in the small intestine of HFD-fed mice. **a** An Oil Red O staining of small intestinal epithelial cells. The intestinal tissues were obtained from 16-week-old NCD-fed or HFD-fed mice. Scale bar, 50 μm. **b**–**e** Proteomic analysis of data from HFD-fed mice^19^, including a gene set enrichment analysis (**b**), heat map of HIF target protein expression (**c**), volcano plot (**d**) and heat map of Golgi-related protein expression (**e**). **f** *Bnip3* mRNA expression. The data are presented as the mean ± standard deviation (*n* = 5 independent experiments), *P < 0.05. **g** A western blot analysis of the expression of the HIF-1α, GM130 and PLIN2 proteins. The protein expression was normalized to that of β-actin and measured via statistical analysis. The data are presented as the mean ± standard deviaiton (*n* = 3 independent experiments), *P < 0.05. For **f** and **g**, the intestinal tissues were obtained from 16-week-old NCD-fed or HFD-fed mice (*n* = 5 independent experiments).

We examined the lipid content in HFD-fed mice to investigate whether lipid accumulation in the small intestine is related to hypoxia. Lipid storage was markedly increased in HFD-fed mice compared with NCD-fed mice (Fig. 1a). This result was confirmed by an increase in PLIN2 expression (Fig. 1g). The proteomic analysis revealed an enriched gene set for hypoxia along with various metabolic pathways, such as fatty acid metabolism and glycolysis (Fig. 1b). In addition, we observed the upregulation of several HIF-inducible proteins, including Tgm2 and Pck1, in HFD-fed mice (Fig. 1c, d). Similarly, the expression of *Bnip3*, a HIF-1α target gene, and the expression of the HIF-1α protein were substantially increased, suggesting that hypoxia is involved in lipid accumulation in the small intestine of HFD-fed mice (Fig. 1f, g). Interestingly, the proteomic analysis of the dataset revealed that the expression of Golgi proteins was markedly reduced in HFD-fed mice compared with NCD-fed mice (Fig. 1d, e). We further confirmed that the expression of GM130, a structural protein of the Golgi apparatus, decreased in HFD-fed mice (Fig. 1g). Taken together, our results suggest that hypoxia induced lipid accumulation and Golgi protein degradation simultaneously in the small intestine of HFD-fed mice.

### Golgi condensation requires the transcriptional activation of HIF-1α upon exposure to hypoxia

**Fig. 2:**
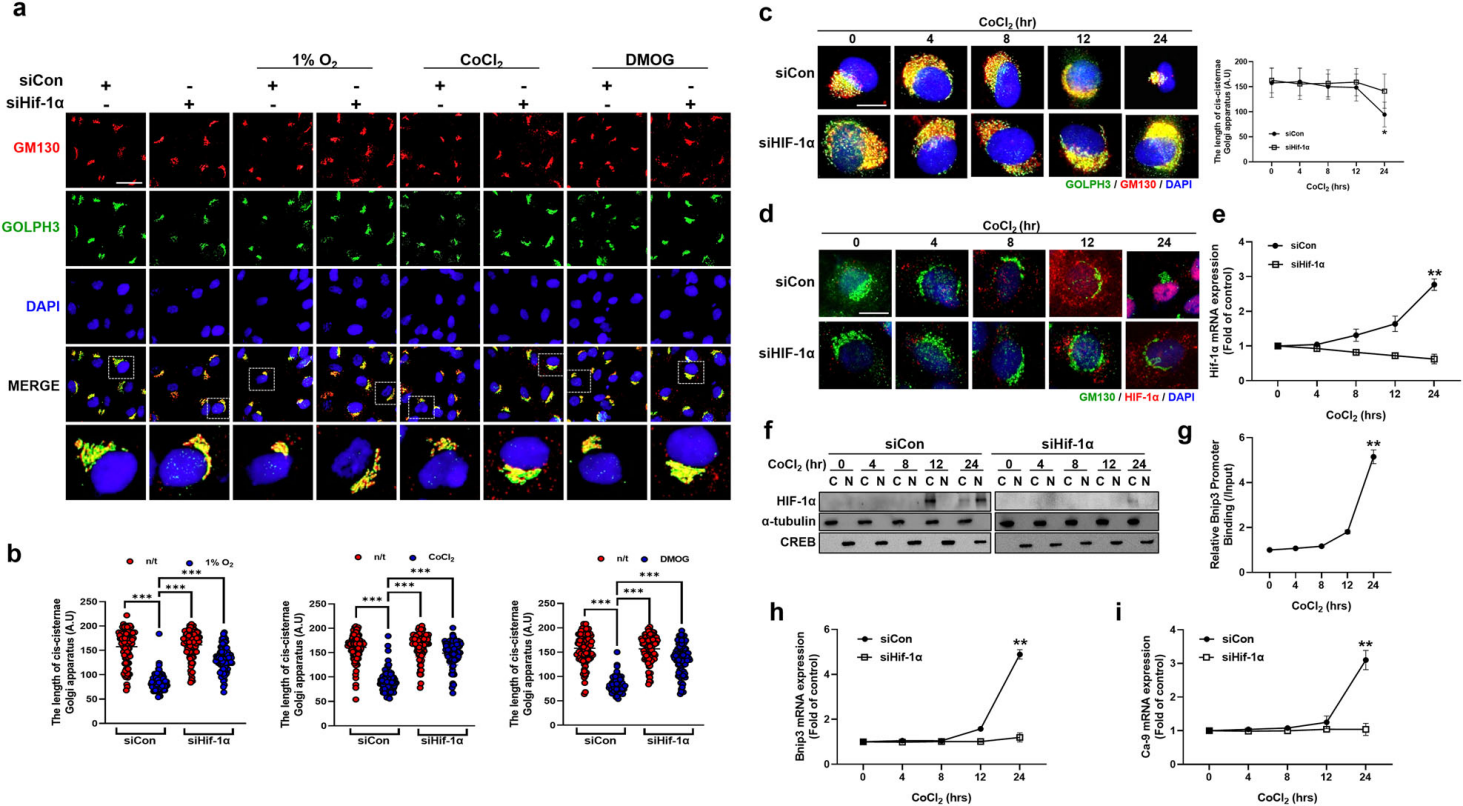
HIF-1α induces Golgi condensation under hypoxic conditions. **a** The protein expression of GM130 and GOLPH3 was measured by immunofluorescence staining. Scale bar, 20 μm, **b** A statistical histogram of the *cis*-Golgi cisternae length. The data are presented as the mean ± standard deviation (s.d.) (*n* = 100), *P < 0.05, **P < 0.01 and ***P < 0.001. The RPE-1 cells were treated with 1% O_2_, CoCl_2_ (200 μM) or DMOG (200 μM) for 24 h after transfection with siHIF-1α. **c**, **d** The protein expression of GOLPH3, GM130 (**c**), GM130 and HIF-1α (**d**) was observed via immunofluorescence staining. Scale bar, 5 μm. The siHIF-1α-transfected cells were treated with CoCl_2_ for the indicated periods. **e** *Hif-1α* mRNA expression. The data are presented as the mean ± standard deviaiton, **P < 0.01. **f** HIF-1α translocation to the nucleus was determined by cell fractionation and immunoblotting for HIF-1α, α-tubulin and cyclic AMP-responsive element binding protein (CREB). **g** A ChIP assay was performed to determine the binding efficiency of HIF-1α to the *Bnip3* promoter region (*n* = 3). **h**, **i** The mRNA expression levels of *Bnip3* (**h**) and *Ca-9* (**i**) were quantified by qRT‒PCR. The data are presented as the mean ± standard (s.d.) (*n* = 3 independent experiments), **P < 0.01.

Since the expression of the Golgi structural protein GM130 decreased, we further tested whether hypoxia induces morphological changes in the Golgi apparatus in RPE-1 cells exposed to three different hypoxic conditions, including a hypoxic chamber with 1% oxygen, CoCl_2_ or DMOG, for 24 h. RPE-1 cells are human retinal pigment epithelial cells widely used to study ciliogenesis and cell division^22^. Recently, they have been commonly used to observe Golgi morphology^23^. Furthermore, they are regarded as an appropriate cell type to study cell death under hypoxic conditions^24^. The length of the *cis*-Golgi cisternae lining the nucleus was measured using an GM130 antibody. The cells were also stained with an antibody against GOLPH3, which localizes to the Golgi apparatus and binds actin to maintain its linear structure. The Golgi apparatus was condensed in cells cultured under the three different hypoxic conditions. In addition, HIF-1α depletion substantially prevented Golgi condensation under hypoxic conditions (Fig. 2a, b).

Golgi condensation occurred 24 h after CoCl_2_ treatment (Fig. 2c). Next, we examined the transcriptional activation of HIF-1α under hypoxic conditions. Along with Golgi condensation, *Hif-1α* mRNA expression increased at 12 h and further intensified at 24 h after CoCl_2_ treatment (Fig. 2e). The intranuclear translocation of HIF-1α from the cytosol was obvious at 24 h of hypoxia exposure (Fig. 2d, f). The transcriptional activity of HIF-1α, which binds to *Bnip3*, was notably increased at 24 h of CoCl_2_ treatment (Fig. 2g). Similarly, the expression of HIF-1α target genes, including *Bnip3* and *Ca-9*, increased (Fig. 2h, i). However, HIF-1α depletion markedly reduced the transcriptional activity of HIF-1α in CoCl_2_-treated cells, which paralleled Golgi condensation.

**Fig. 3:**
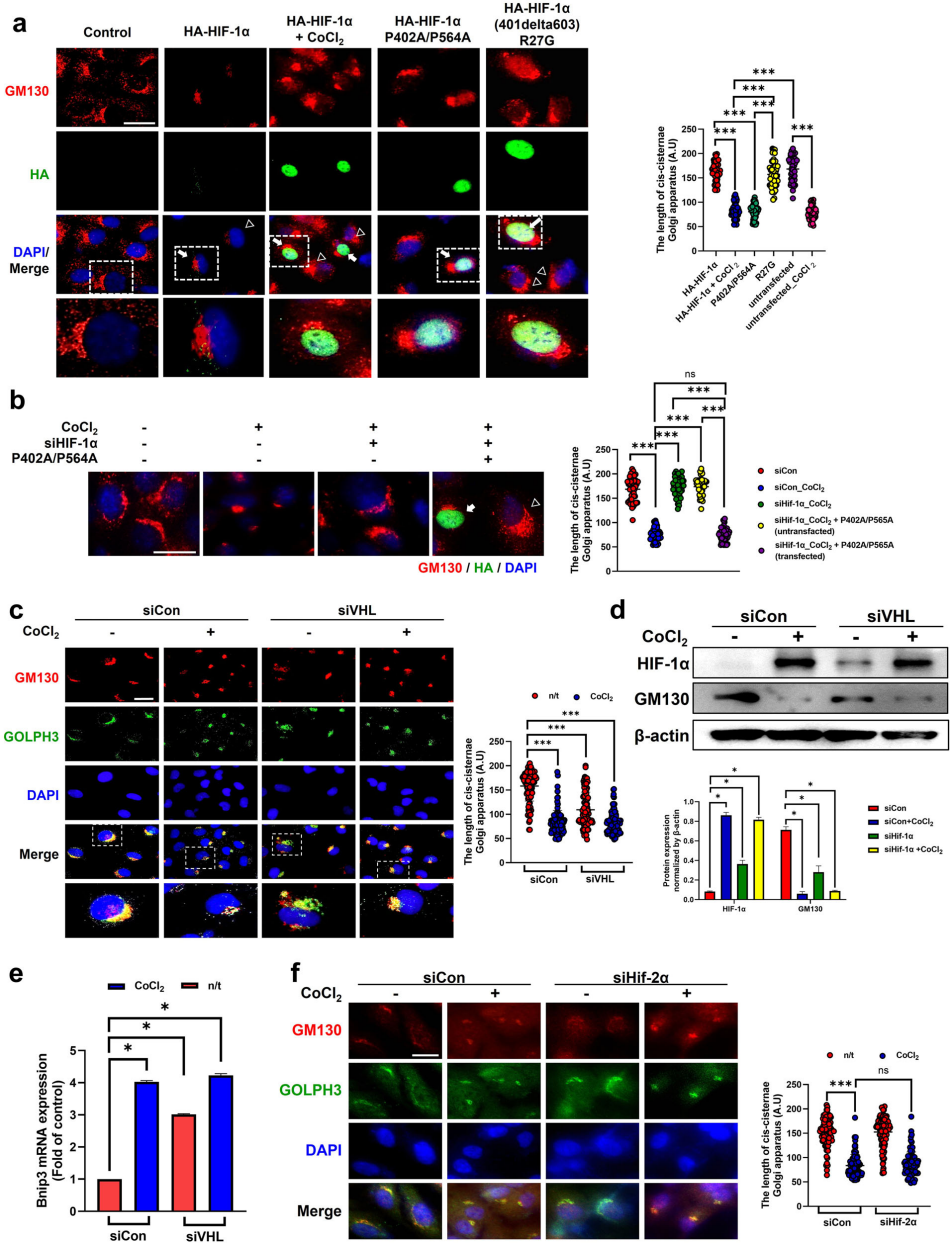
The transcriptional activity of HIF-1α is required for Golgi condensation. **a**–**c** The protein expression of GM130, HA (**a**, **b**) and GM130 and GOLPH3 (**c**) was observed using immunofluorescence staining. For **b** the arrows and triangles represent either plasmid-transfected cells or untransfected cells, respectively. Scale bar, 20 μm. A statistical histogram of the *cis*-Golgi cisternae length is shown. The data are presented as the mean ± standard deviation (s.d.) (*n* = 100), ***P < 0.001. **d** The protein expression of HIF-1α, GM130 and β-actin, as determined by immunoblotting. The protein expression was normalized to that of β-actin and measured via statistical analysis. The data are presented as the mean ± s.d. (*n* = 3 independent experiments), *P < 0.05. **e** *Bnip3* mRNA expression was quantified by qRT‒PCR. The data are presented as the mean ± s.d. (*n* = 4 independent experiments), *P < 0.05. For **a** and **b**, RPE-1 cells were transfected with plasmids expressing various HIF-1α mutants, and for **c** and **e**, the cells were transfected with siVHL. **f** The cells were transfected with siHIF-2α, and the expression of the GM130 and GOLPH3 proteins was measured by immunofluorescence staining. Scale bar, 10 μm. A statistical histogram of the *cis*-Golgi cisternae length is shown. The data are presented as the mean ± s.d. (*n* = 100), ***P < 0.001.

We used two different plasmids to further evaluate the effect of the transcriptional activity of HIF-1α on Golgi condensation (Supplementary Fig. 1a, b). HA-HIF-1α (P402A/P564A) was mutated at sites 402 and 564, allowing it to function as a transcription factor without being degraded by the proteasome. HA-HIF-1α (R27G) was mutated at the 27th amino acid, with a deletion from amino acids 401–603, and the protein was neither degraded nor bound the target gene. HA-HIF-1α (P402A/P564A)-transfected cells showed obvious translocation of HIF-1α to the nucleus along with Golgi condensation in the absence of CoCl_2_. However, HA-HIF-1α (R27G)-transfected cells exhibited HIF-1α translocation to the nucleus without Golgi condensation (Fig. 3a). Although HIF-1α depletion restored the Golgi structure to a flattened shape in the presence of CoCl_2_, transfection with HA-HIF-1α (P402A/P564A) resulted in no conversion to a flat Golgi apparatus (Fig. 3b).

We next confirmed the effect of HIF-1α on Golgi condensation by transfecting an siRNA targeting Von Hippel‒Lindau (VHL). VHL depletion using siVHL transfection promoted Golgi condensation by protecting against HIF-1α protein degradation in cells that were not treated with CoCl_2_ (Fig. 3c, d). Furthermore, siVHL transfection upregulated *Bnip3* expression in cells treated with and without CoCl_2_ (Fig. 3e). However, compared with HIF-1α depletion, the depletion of HIF-2α did not prevent Golgi condensation in CoCl_2_-treated cells (Fig. 3f). These results suggest that the transcriptional activity of HIF-1α is critically necessary for Golgi condensation under hypoxic conditions.

### Proteasomal degradation of GM130 plays a key role in Golgi condensation upon hypoxia exposure

**Fig. 4:**
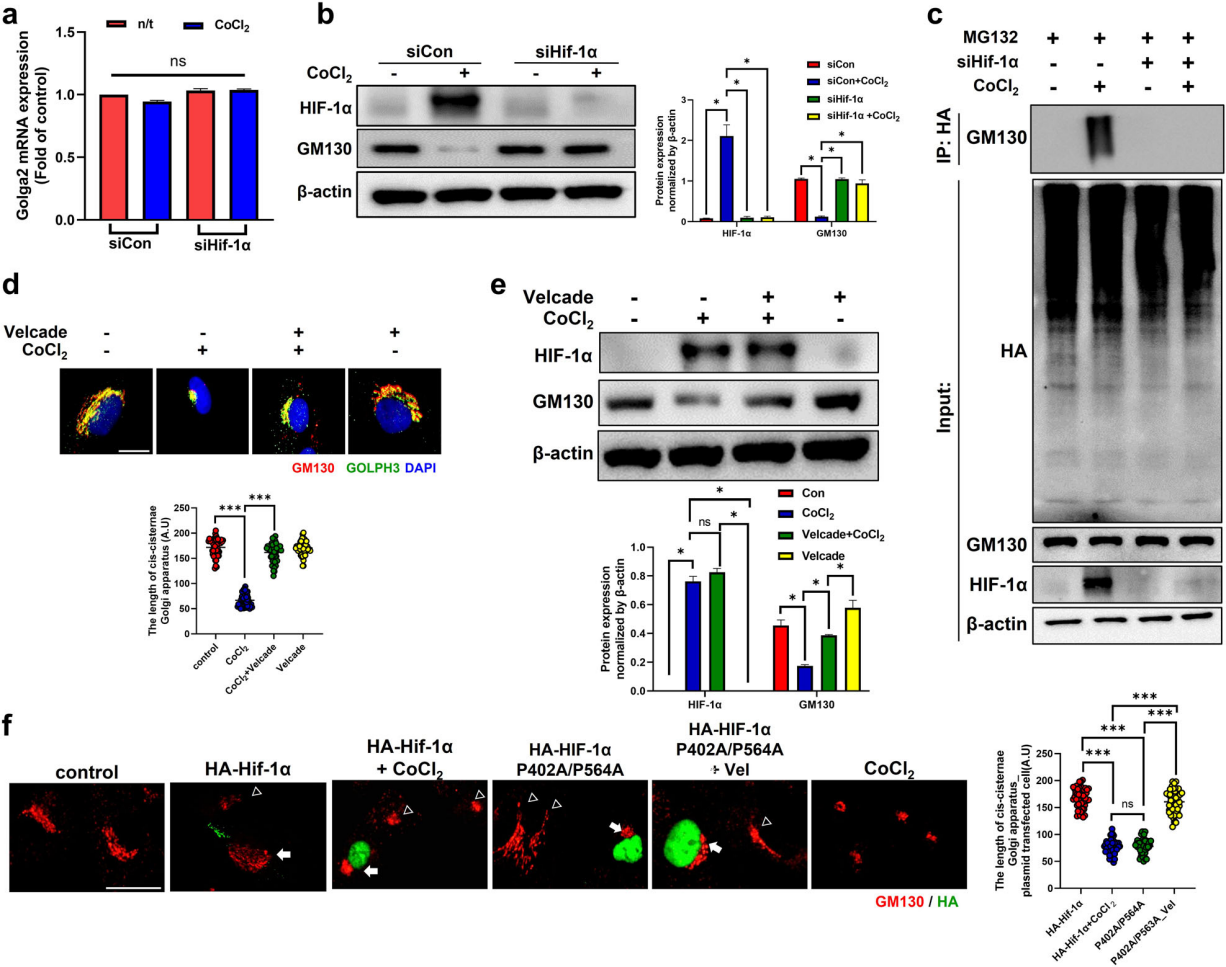
Proteasomal degradation of GM130 causes Golgi condensation under hypoxic conditions. **a** The expression of the *Golga2* mRNA was quantified by qRT‒PCR. RPE-1 cells were transfected with siRNAs, treated with CoCl_2_ for 12 h and then supplemented with either the proteasomal inhibitor MG132 or the 26S proteasome inhibitor Velcade. The data are presented as the mean ± standard deviation (s.d.) (*n* = 3 independent experiments). **b** The protein expression of GM130, HIF-1α and β-actin was determined by immunoblotting. The protein expression was normalized to that of β-actin and was measured via statistical analysis. The data are presented as the mean ± s.d. (*n* = 3 independent experiments), *P < 0.05. **c** IP of HA-tagged GM130 and immunoblotting for GM130, HIF-1α and β-actin. **d** The protein expression of GM130 and GOLPH3 was observed by immunofluorescence staining. Scale bar, 5 μm. A statistical histogram of the *cis*-Golgi cisternae length is shown. The data are presented as the mean ± s.d. (*n* = 100), ***P < 0.001. **e** The protein expression of HIF-1α, GM130 and β-actin was determined by immunoblotting. The protein expression was normalized to that of β-actin and was measured via statistical analysis. The data are presented as the mean ± s.d. (*n* = 3 independent experiments), *P < 0.05. **f** The expression of the GM130 and HA proteins was measured by immunofluorescence staining. The arrows and triangles represent either plasmid-transfected cells or untransfected cells, respectively. Scale bar, 10 μm. A statistical histogram of the *cis*-Golgi cisternae length is shown. The data are presented as the mean ± s.d. (*n* = 100), ***P < 0.001.

We measured the GM130 mRNA and protein expression levels. The expression of the *Golga2* mRNA did not change in CoCl_2_-treated cells, irrespective of the presence of siHIF-1α (Fig. 4a). However, the GM130 protein level decreased upon CoCl_2_ treatment, which was reversed by siHIF-1α, indicating that HIF-1α depletion prevented GM130 degradation (Fig. 4b). The cells were transfected with HA-ubiquitin to confirm the proteasomal degradation of GM130. GM130 ubiquitination was increased by CoCl_2_ treatment, whereas it was markedly decreased by siHIF-1α, regardless of CoCl_2_ treatment (Fig. 4c). Furthermore, treatment with Velcade, a 26S proteasome inhibitor, suppressed GM130 degradation in CoCl_2_-treated cells (Fig. 4d, e). Golgi condensation in HA-HIF-1α (P402A/P564A)-transfected cells was attenuated by Velcade, suggesting the involvement of proteasomal degradation in HIF-1α-mediated Golgi condensation (Fig. 4f). These results suggest that GM130, which is crucial for maintaining the structure of the Golgi apparatus, undergoes ubiquitination and subsequent proteasomal degradation under hypoxic conditions.

### The E3 ligase NEDD4 degrades GM130 in the Golgi apparatus under hypoxic conditions

**Fig. 5:**
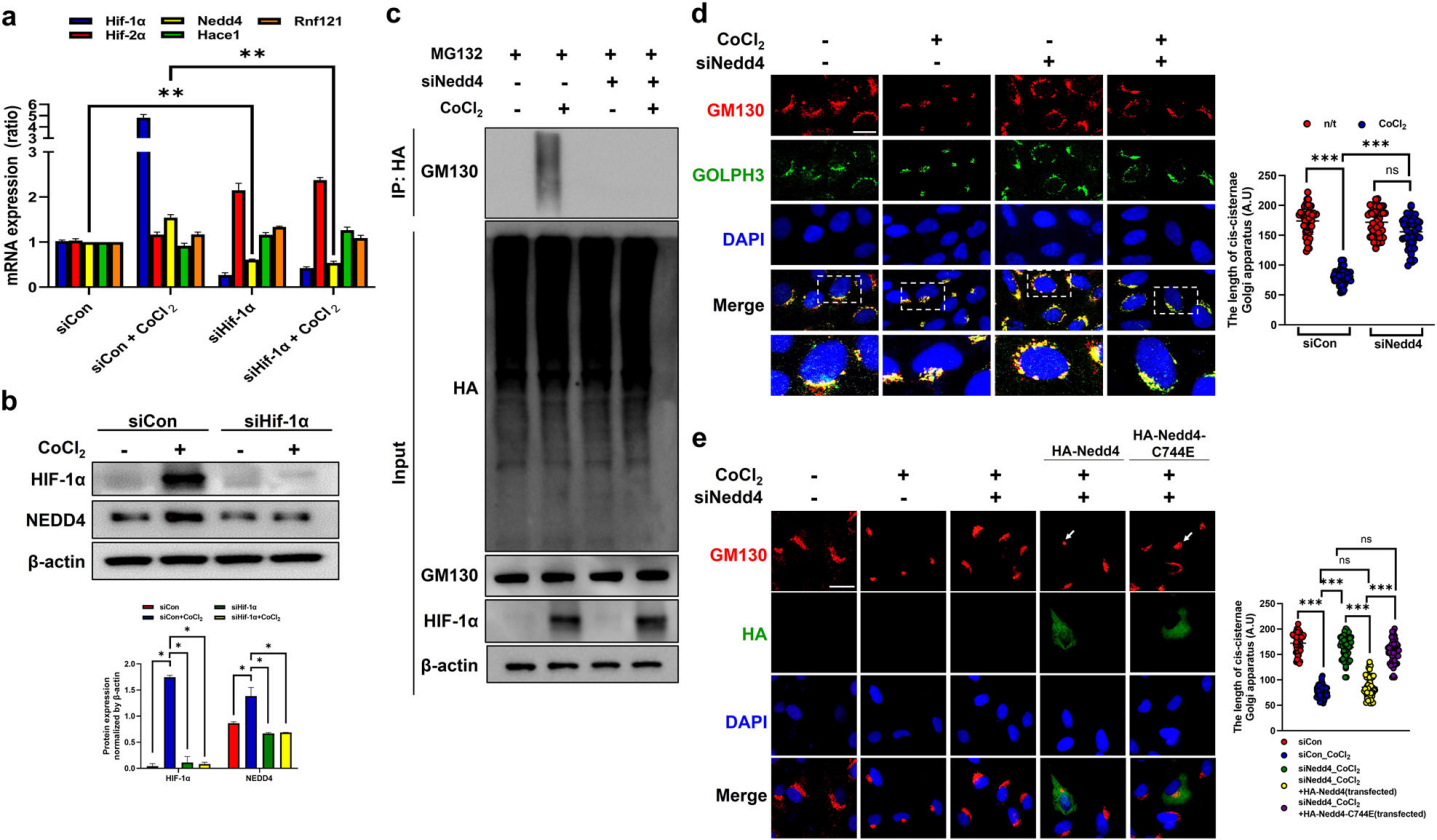
NEDD4 induces GM130 ubiquitination during Golgi condensation caused by hypoxia. **a** The mRNA expression was quantified by qRT‒PCR. The data are presented as the mean ± standard deviation (s.d.) (*n* = 3 independent experiments), *P < 0.05. **b** The protein expression of HIF-1α, NEDD4 and β-actin was determined by immunoblotting. The protein expression was normalized to that of β-actin and was measured via statistical analysis. The data are presented as the mean ± s.d. (*n* = 3 independent experiments), *P < 0.05. For **a** and **b**, the RPE-1 cells were transfected with siHIF-1α and then treated with CoCl_2_. **c** siNEDD4-transfected cells were processed for IP of HA-tagged GM130 and immunoblotting for GM130, HIF-1α and β-actin. **d** The protein expression of GM130 and GOLPH3 was measured by immunofluorescence staining. Scale bar, 20 μm. A statistical histogram of the *cis*-Golgi cisternae length is shown. The data are presented as the mean ± s.d. (*n* = 100), ***P < 0.001. **e** Nedd4-depleted cells were transfected with HA-Nedd4 or HA-Nedd4-C744E and then treated with CoCl_2_. The protein expression of GM130 and HA was observed by immunofluorescence staining. The arrows indicate the Golgi apparatus in the transfected cells. Scale bar, 20 μm. A statistical histogram of the *cis*-Golgi cisternae length is shown. The data are presented as the mean ± s.d. (*n* = 100), ***P < 0.001.

We examined whether E3 ligases are responsible for the proteasomal degradation of GM130 under hypoxic conditions by analyzing RNA-seq data from 501mel cells exposed to hypoxia at different time points^25^. E3 ligases in the Golgi apparatus include *Hace1*, *Rnf121* and *Nedd4* (refs. ^26–28^). Among these genes, *Nedd4* expression was modified in the absence of HIF-1α (Supplementary Fig. 2a, b). Both the mRNA and protein expression levels of NEDD4 increased in CoCl_2_-treated cells, changes that were attenuated by HIF-1α depletion, irrespective of CoCl_2_ treatment (Fig. 5a, b).

We confirmed that NEDD4 depletion (Supplementary Fig. 2c) reduced GM130 ubiquitination and protected against Golgi condensation in CoCl_2_-treated cells (Fig. 5c, d). Furthermore, the E3 ligase activity of NEDD4 was evaluated by transfecting NEDD4-depleted cells with either HA-Nedd4 or HA-Nedd4-C744E (Supplementary Fig. 2d) and treating them with CoCl_2_. Golgi condensation was reduced in siNEDD4-transfected cells in the presence of CoCl_2_ (Fig. 5e). However, siNEDD4 did not ameliorate Golgi condensation induced by CoCl_2_ in HA-Nedd4-transfected cells compared with siNEDD4-transfected cells. In contrast, siNEDD4 kept ameliorating Golgi condensation in HA-Nedd4 C744E-transfected cells, losing the E3 ligase domain (Fig. 5e). These results indicate that NEDD4 plays a pivotal role in the proteasomal degradation of GM130 during hypoxia-induced Golgi condensation.

### Golgi condensation causes lipid accumulation in the small intestine under hypoxic conditions

**Fig. 6:**
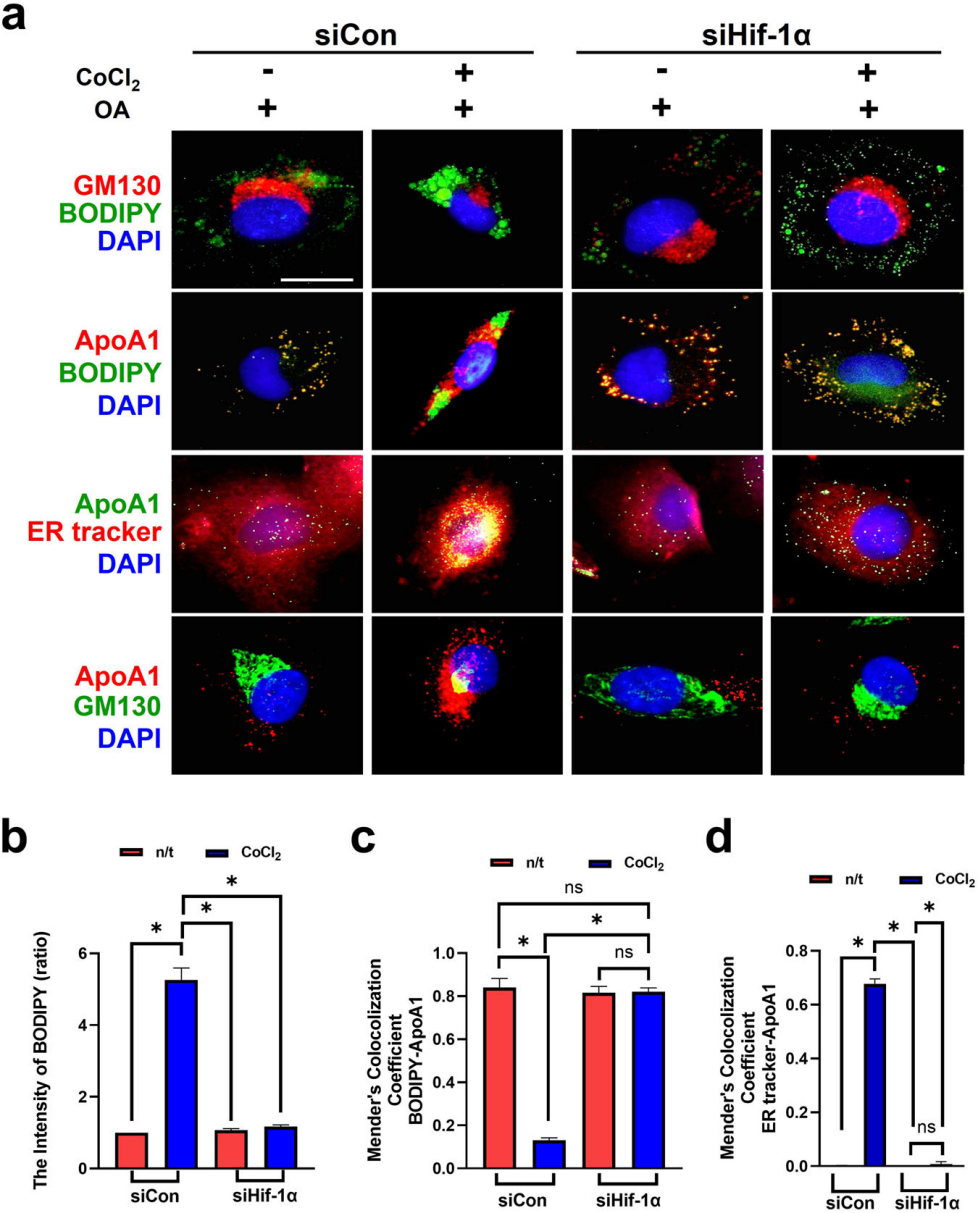
HIF-1α depletion prevents the condensation of the Golgi structure and decreases both lipid accumulation and AopA1 retention in Fhs74Int primary intestinal epithelial cells treated with CoCl_2_. **a** Immunofluorescence staining for GM130 and BODIPY was performed to visualize the Golgi and lipids; ApoA1 was used as a marker for lipoproteins (chylomicron/HDL), the ER tracker was used as a marker for the ER and DAPI was used as a marker for the nuclei. Scale bar, 5 μm. **b**–**d** The statistical histograms of the BODIPY intensity (**b**), Mender’s colocalization coefficient of BODIPY-APOA1 (**c**) and Mender’s colocalization coefficient of the ER tracker APOA1 (**d**). The data are presented as the mean ± standard deviation (*n* = 5 independent experiments), *P < 0.05.

HIF-1α plays a major role in regulating metabolism and influences metabolic diseases such as obesity^29^. Hypoxia, which has been reported in patients with obesity, results in lipid accumulation in small intestinal enterocytes^29^. Therefore, we examined whether Golgi condensation causes lipid accumulation through HIF-1α activation in Fhs74Int primary intestinal epithelial cells. Golgi condensation manifested as a reduced GM130 intensity and increased BODIPY intensity (Fig. 6a, b). Furthermore, the intensity of the lipoprotein (chylomicron/HDL) marker apolipoprotein A1 (ApoA1) increased with increasing BODIPY intensity during GM130 degradation (Fig. 6a, c). As ApoA1 is synthesized in the ER and secreted into the Golgi apparatus through a nonprechylomicron transport vesicle^1^, we confirmed that the increased intensity of ApoA1 colocalized with the ER tracker (Fig. 6a, d). ApoA1 retention was consistently observed with a reduced GM130 intensity (Fig. 6a). However, siHIF-1α transfection suppressed the increased intensity of ApoA1 and BODIPY in CoCl_2_-treated cells (Fig. 6a, b). We also confirmed these phenotypes in another type of epithelial cell, Caco-2 cells. In Caco-2 cells, the GM130 intensity was reduced, and the length of the cisternae was notably decreased following CoCl_2_ treatment (Supplementary Fig. 3a). Moreover, the BODIPY intensity was increased by CoCl_2_ treatment in these cells (Supplementary Fig. 3a). However, both phenotypes were successfully rescued by siHIF-1α transfection, consistent with the results observed in Fhs74int cells (Supplementary Fig. 3a).

We confirmed whether Golgi condensation increases the retention of lipids and ApoA1 by transfecting siGolph3 (Supplementary Fig. 3b)^30^. Golph3 depletion increased Golgi condensation and the intensity of BODIPY and ApoA1, whereas it reduced GM130 expression, irrespective of CoCl_2_ treatment (Supplementary Fig. 3c). We also evaluated whether other lipid metabolism-related proteins might be involved in Golgi condensation and measured the levels of several lipid-related markers, including DGAT-1, I-FABP and ACOX-1 (Supplementary Fig. 3d). The expression of these proteins was increased by CoCl_2_ treatment; however, a clear difference was not observed between the control and HIF-1α depletion groups. These results indicate that other lipid-related pathways might not be associated with HIF-1α signaling. Overall, we propose that Golgi condensation promotes lipid accumulation and impairs ApoA1 trafficking to the Golgi apparatus in small intestinal epithelial cells under hypoxic conditions.

### The enzymatic activity of NEDD4 is pivotal for lipid accumulation and ApoA1 retention in intestinal epithelial cells under hypoxic conditions

**Fig. 7:**
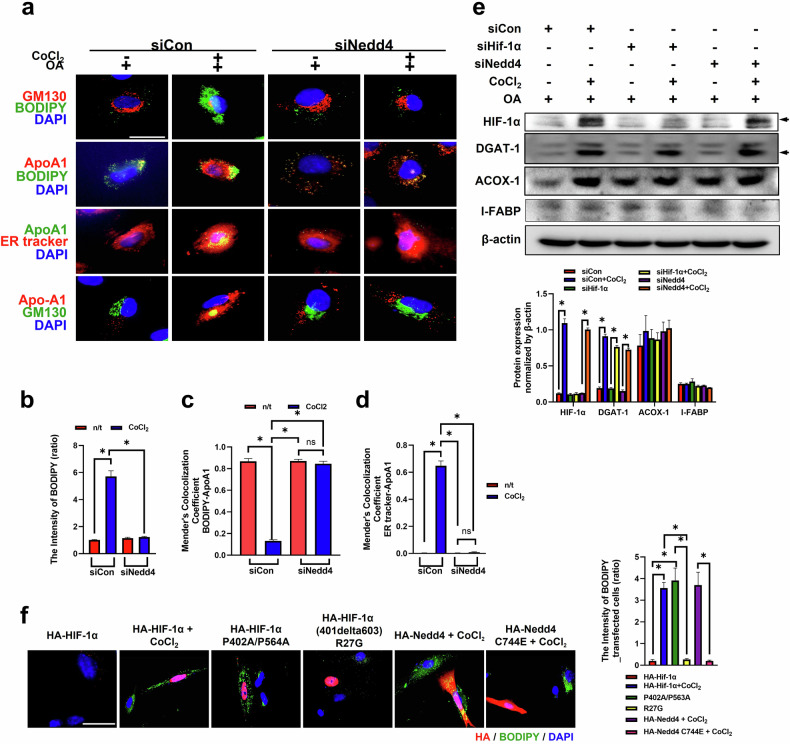

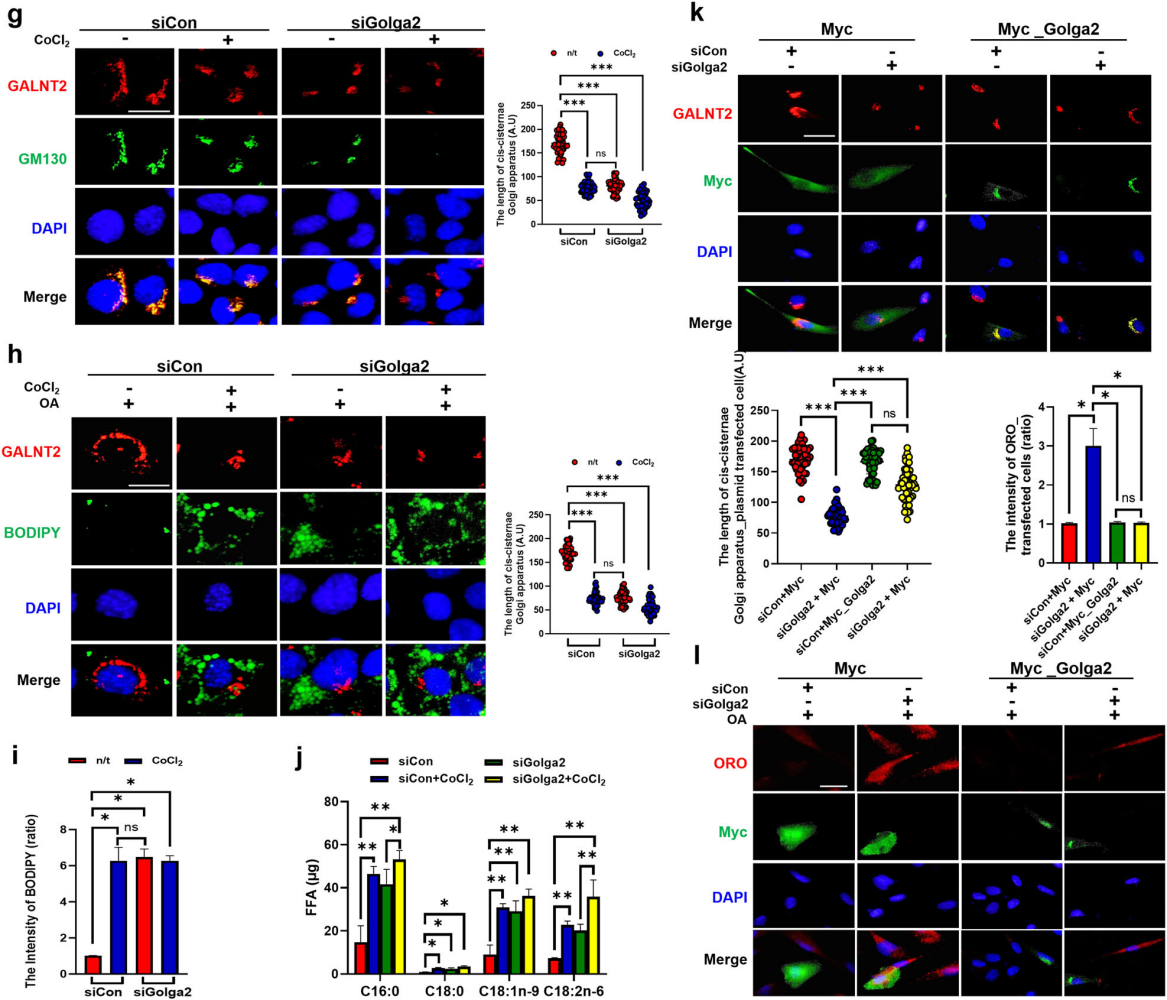
GM130 degradation increases Golgi condensation and lipid accumulation via NEDD4 under hypoxic conditions. **a** Immunofluorescence staining for GM130 and BODIPY was performed to visualize the Golgi apparatus and lipids, respectively. ApoA1 was used as a marker for lipoproteins (chylomicron/HDL), the ER tracker was used as a marker for the ER and DAPI was used as a marker for the nuclei. Scale bar, 5 μm. Statistical histograms of the BODIPY intensity (**b**), Mender’s colocalization coefficient of BODIPY-APOA1 (**c**) and Mender’s colocalization coefficient of the ER tracker APOA1 (**d**) are shown. The data are presented as the means ± standard deviation (s.d.) (*n* = 5 independent experiments), *P < 0.05. For **a**–**d**, the Fhs74Int cells were transfected with either siNEDD4 or siHIF-1α before treatment with oleic acid (OA) and CoCl_2_. **e** The protein expression of HIF-1α, DGAT-1, ACOX1, I-FABP and β-actin was determined by immunoblotting. The protein expression was normalized to that of β-actin and was measured via statistical analysis. The data are presented as the mean ± s.d. (*n* = 3 independent experiments), *P < 0.05. **f** Fhs74Int cells were transfected with the indicated plasmids and treated with CoCl_2_. Immunofluorescence staining for HA and BODIPY was performed to visualize the transfected cells and lipids. Scale bar, 10 μm. The intensity of BODIPY in the transfected cells was statistically analyzed. The data are presented as the mean ± s.d. (*n* = 5 independent experiments), *P < 0.05. **g**–**i** Fhs74int cells were transfected with either siCon or siGolga2 (**g**) before treatment with OA and CoCl_2_ (**h** and **i**). **g** Immunofluorescence staining for GALANT2 and GM130 was performed to visualized the Golgi apparatus. A statistical histogram of the cis-Golgi cisternae length is shown. The data are presented as the mean ± s.d. (*n* = 100), ***P < 0.001. **h** Immunofluorescence staining for GALNT2 and BODIPY was performed to visualize the Golgi apparatus and lipids, respectively. A statistical histogram of the cis-Golgi cisternae length is shown. The data are presented as the mean ± s.d. (*n* = 100), ***P < 0.001. **i** A statistical histogram of the BODIPY intensity. The data are presented as the mean ± s.d. (*n* = 5 independent experiments), *P < 0.05. **j** The concentration of free fatty acids was measured via GC‒MS. The data are presented as the mean ± s.d. (*n* = 5 independent experiments), *P < 0.05 and **P < 0.01. **k** A statistical histogram of the *cis*-Golgi cisternae length. The data are presented as the mean ± s.d. (*n* = 100), ***P < 0.001. **l** Transfected cells treated with OA. A statistical histogram of the ORO intensity in transfected cells is shown. The data are presented as the mean ± s.d. (*n* = 5 independent experiments), *P < 0.05. Fhs74int cells were transfected with either siCon or siGolga2 before being transfected with either Myc or Myc_Golga2. Immunofluorescence staining for GALNT2 and BODIPY was performed to visualize the Golgi apparatus and lipids, respectively. Green Fluorescent Protein (GFP) indicates plasmid-transfected cells. Scale bar, 20 μm.

We found that NEDD4 played a key role in degrading GM130 under hypoxic conditions, which subsequently induced Golgi condensation. Therefore, we investigated whether NEDD4 affects lipid accumulation in Fhs74int cells under hypoxic conditions. Golgi condensation, lipid accumulation and ApoA1 retention in the ER were consistently observed after CoCl_2_ treatment (Fig. 7a, c). However, NEDD4 depletion resulted in reduced BODIPY and ApoA1 intensities and maintained the linear structure of the Golgi apparatus (Fig. 7a–d). No difference in the expression of lipid-related proteins, such as DGAT-1, I-FABP and ACOX-1, was observed between the siControl- and siNEDD4-transfected groups (Fig. 7e).

We next evaluated the effects of the enzymatic activities of HIF-1α and NEDD4 on lipid accumulation, ApoA1 retention and Golgi condensation under hypoxic conditions. Compared with nontransfected cells, HA-HIF-1α (P402A/P564A)-transfected cells presented greater lipid accumulation. HA-HIF-1α (R27G)-transfected cells did not exhibit lipid accumulation (Fig. 7f). In addition, HA-NEDD4-C744E-transfected cells, which previously exhibited Golgi condensation, presented reduced lipid accumulation compared with nontransfected cells or HA-NEDD4-transfected cells (Fig. 7f). These results illustrate that the NEDD4-regulated cascade of lipid accumulation and ApoA1 trafficking to the Golgi apparatus is markedly impaired under hypoxic conditions. In summary, the enzymatic activities of both HIF-1α and NEDD4 may be pivotal in Golgi condensation, which further exacerbates lipid accumulation under hypoxic conditions.

### GM130 depletion promotes Golgi condensation and lipid accumulation in intestinal epithelial cells

We transfected Fhs74int cells with siGolga2 to reduce GM130 expression and investigate the roles of GM130 degradation in Golgi condensation and lipid accumulation (Supplementary Fig. 3e). The length of the Golgi apparatus was reduced in cells transfected with siGolga2, as indicated by the expression of GALNT2, a functional Golgi protein, regardless of CoCl_2_ treatment (Fig. 7g). In addition, GM130 depletion led to an increase in the BODIPY intensity, which was independent of CoCl_2_ treatment (Fig. 7h, i). This observation suggests that Golgi condensation contributes to lipid accumulation.

We profiled various free fatty acids to determine the types of free fatty acid that accumulated due to Golgi condensation and found that the concentrations of C16:0 (palmitic acid) and C18:2n-6 (linoleic acid) were substantially increased when siGolga2 transfection was combined with CoCl_2_ treatment (Fig. 7j).

We overexpressed GM130 in siGolga2-transfected cells, which were then transfected with either Myc or Myc_Golga2, to further assess the importance of GM130 in Golgi condensation and lipid accumulation. We observed that Myc transfection reduced the length of the cisternae in siGolga2-transfected cells. However, Myc_Golga2 transfection prevented Golgi condensation in siGolga2-transfected cells (Fig. 7k). Moreover, Myc transfection increased the ORO intensity in siGolga2-transfected cells, whereas Myc_Golga2 transfection decreased the ORO intensity, even in cells transfected with siGolga2 (Fig. 7l). Together, these results indicate that GM130 depletion promotes lipid accumulation, a process modulated by Golgi condensation.

### Pharmacologic inhibition of HIF-1α prevents GM130 degradation and lipid accumulation in the small intestine of HFD-fed mice

**Fig. 8:**
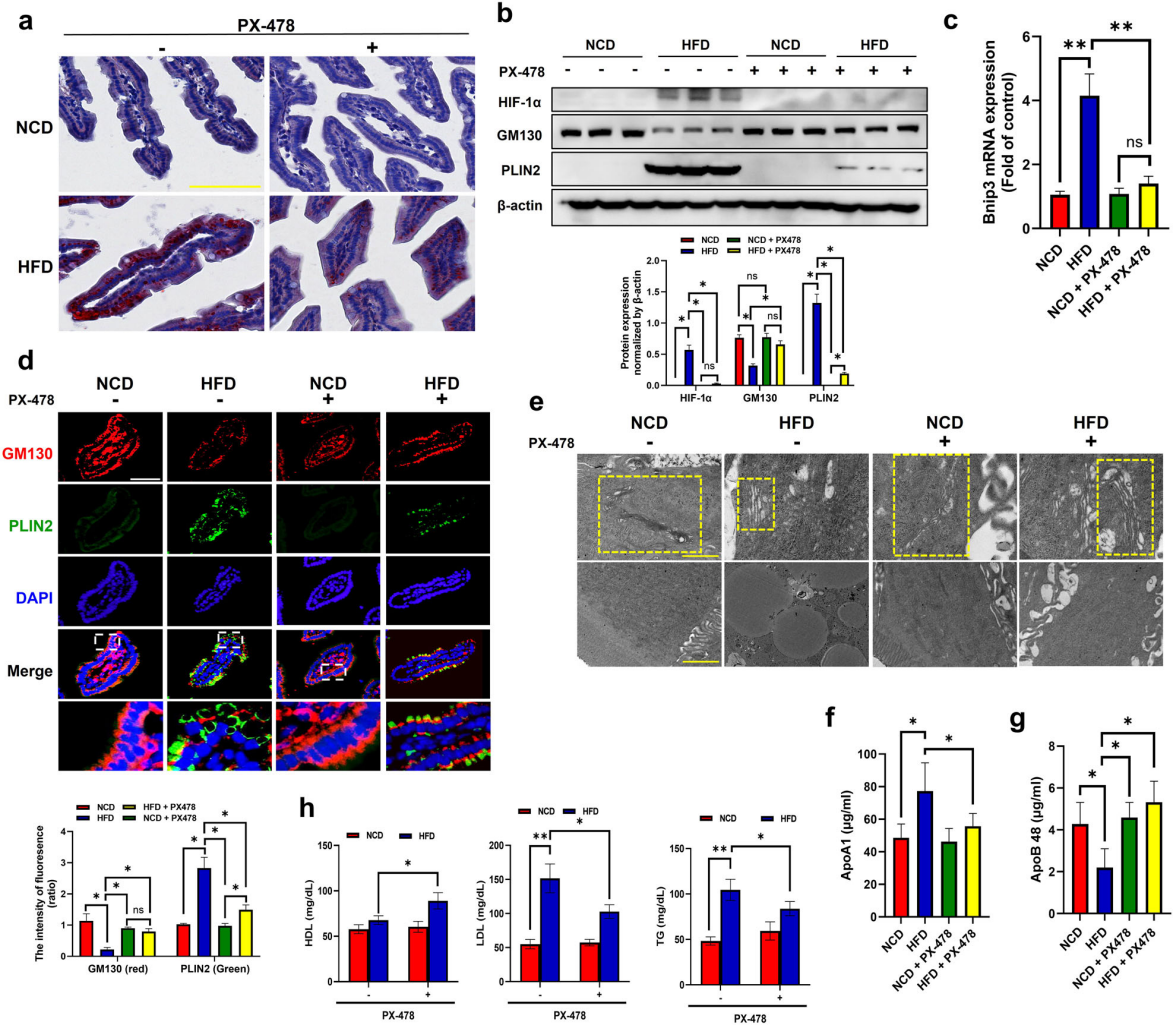
Inhibition of HIF-1α attenuates hypoxia-mediated GM130 degradation and lipid accumulation in the small intestine of HFD-fed mice. **a** Oil Red O staining of small intestinal epithelial cells. Eight-week-old mice were fed either a NCD or HFD for 16 weeks. PX-478 (10 mg/kg) was administered via oral gavage three times a week starting at 6 weeks of HFD feeding. Scale bar, 50 μm. **b** The protein expression of HIF-1α, GM130, PLIN2 and β-actin was measured by immunoblotting. The protein expression was normalized to that of β-actin and was measured via statistical analysis. The data are presented as the mean ± standard deviation (s.d.) (*n* = 3 independent experiments), *P < 0.05. **c** *Bnip3* mRNA expression was quantified by qRT‒PCR. The data are presented as the mean ± s.d. (*n* = 5 independent experiments), *P < 0.05 and **P < 0.01. **d** The protein expression of GM130 and PLN2 was observed by immunofluorescence staining. Scale bar, 50 μm. A statistical histogram of the fluorescence intensity is shown. The data are presented as the mean ± s.d. (*n* = 5 independent experiments), *P < 0.05. **e** TEM images of the Golgi apparatus in intestinal epithelial cells. Scale bar, 2 μm (top), 500 nm (bottom). **f** The ApoA1 concentration in the intestinal jejunum tissue was measured using an ELISA. The data are presented as the mean ± s.d. (*n* = 5 independent experiments), *P < 0.05. **g** The ApoB48 concentration in the serum was measured using an ELISA after treatment with 10 μl/g (body weight) lard oil. The data are presented as the means ± s.d. (*n* = 5 independent experiments), *P < 0.05. **h** The serum LDL and TG levels were determined using ELISAs. The data are presented as the mean ± s.d. (*n* = 5 independent experiments), *P < 0.05 and **P < 0.01.

We administered the HIF-1α inhibitor PX-478 to HFD-fed mice to confirm that hypoxia-induced Golgi condensation leads to intestinal lipid accumulation through HIF-1α-mediated GM130 ubiquitination. Lipid storage in the small intestine was markedly increased in HFD-fed mice (Fig. 8a). Conversely, the ORO staining intensity was reduced in PX-478-treated HFD-fed mice (Fig. 8a). In HFD-fed mice, GM130 expression decreased and the expression of HIF-1α and PLIN2 increased. However, compared with HFD-fed mice, PX-478 treatment prevented GM130 degradation, decreased PLIN2 expression and downregulated HIF-1α expression (Fig. 8b). In contrast to GM130 degradation, the expression of other Golgi structural proteins, such as Golgin45, GRASP55 and GRASP65, did not change under HFD conditions, regardless of PX-478 treatment (Supplementary Fig. 4a). The pharmacological effect of PX-478 on HIF-1α transcriptional activity was confirmed by *Bnip3* mRNA expression in HFD-fed mice (Fig. 8c). The GM130 intensity was also decreased in the mice and restored to the control level in the PX-478-treated HFD-fed mice (Fig. 8d). The PLIN2 intensity was associated with a reduced number of lipid droplets (LDs) in the small intestine of PX-478-treated HFD-fed mice, indicating that HIF-1α activation is required for GM130 degradation and lipid accumulation in small intestinal cells both in vivo and in vitro.

We also determined the effects of pharmacologic inhibition of HIF-1α on structural modifications of the Golgi apparatus under HFD conditions and visualized the Golgi apparatus in mouse intestinal epithelial cells using TEM. In the epithelial cells of the NCD-fed mice, the Golgi apparatus displayed a linear structure, whereas in the HFD-fed mice, the Golgi apparatus was shortened with lipid accumulation. However, both Golgi condensation and lipid accumulation were prevented in HFD-fed mice treated with PX-478 (Fig. 8e).

Next, we measured the amount of ApoA1 in the small intestine of the mice to investigate its function. Similar to the results obtained in Fhs74Int cells, ApoA1 retention was prominent in HFD-fed mice, whereas PX-478 treatment notably decreased ApoA1 retention in HFD-fed mice (Fig. 8f). We also measured the concentration of ApoB48, a chylomicron marker, in mouse serum. It was substantially decreased in HFD-fed mice, whereas this reduction was prevented in HFD-fed mice treated with PX-478 (Fig. 8g). Chylomicron secretion was further assessed using a Transwell system^31^. The Caco-2 cells were seeded into the insert compartment, and the media were collected from the apical (A) and basolateral (B) sections to measure the ApoB concentration (Supplementary Fig. 4b). In section A, the ApoB concentration did not change, whereas in section B, it was notably reduced by CoCl_2_ treatment (Supplementary Fig. 4b). However, this decrease was prevented by PX-478 treatment (Supplementary Fig. 4b). Moreover, the HDL level, which was decreased in HFD-fed mice, was substantially restored by PX-478 administration (Fig. 8h). The metabolic phenotype, including reduced serum levels of LDL and TG, was significantly improved in PX-478-treated HFD-fed mice compared with mice fed the HFD alone (Fig. 8h). These results highlight that pharmacologic inhibition of HIF-1α with PX-478 not only prevents ApoA1 retention in the small intestine for lipoprotein maturation but also improves metabolic phenotypes, including increased HDL levels and decreased LDL and TG levels, in vivo.

## Discussion

The structure of the Golgi apparatus has a notable effect on intracellular lipid trafficking. Perturbation of the Golgi structure results in lipid accumulation and potentially impacts lipoprotein secretion, culminating in reduced levels of HDL^32^. These findings collectively underscore that structural modifications of the Golgi apparatus can impede lipid trafficking while concurrently influencing lipoprotein secretion from small intestinal epithelial cells^2^. Among the various factors contributing to structural alterations in the Golgi apparatus, hypoxia is a significant condition capable of inducing substantial changes in both the shape and function of the Golgi apparatus^9^. The precise manner in which hypoxia influences the Golgi structure and lipid trafficking in the small intestine remains unexplored. Although activation of the HIF family represents a protective mechanism against low-oxygen environments^33^, prolonged HIF activation can paradoxically lead to hypoxic stress. These stresses can ultimately cause adipocyte dysfunction^34^, compromise thermogenic capacity^35^ and curtail fatty acid β-oxidation^36^. Several studies have reported the therapeutic potential of HIF inhibitors for metabolic disorders^37^. Excessive lipid accumulation was observed in the small intestine of the HFD-fed model. We established a HFD-induced obesity model to determine whether hypoxia-mediated Golgi condensation causes impaired lipid trafficking in the small intestine. Compared with that of the NCD-fed mice, the small intestine of the HFD-fed mice presented greater lipid accumulation in epithelial cells. Lipid accumulation in epithelial cells may result in excessive lipid absorption after the consumption of a HFD. However, overnight fasting after the consumption of a HFD did not reduce the number of LDs in the intestine. The trafficking, utilization and secretion of lipids are much faster in the intestine than in other tissues, indicating that lipid accumulation in the small intestine is not an ordinary phenotype^1^. Lipid accumulation in epithelial cells suggests that lipid trafficking and utilization may be impaired in HFD-fed or obese models. Among several factors that impair lipid trafficking in the small intestine of HFD-fed mice, hypoxia might be a critical factor that results in constant stress to epithelial cells^38^. Hypoxia occurs in several tissues, including adipose tissue and the intestine^13,39^. The proteomic results revealed increased expression of HIF-1α target proteins rather than HIF-2α. However, the expression of HIF-2α was higher than that of HIF-1α in ileum biopsies from patients with obesity^13^. HIF-1α and HIF-2α exhibit structural similarities; both share essential domains, such as the helix-loop-helix domain, Per-Arnt-SIM domain, N/C-terminal transactivation domain, inhibitory domain and oxygen-dependent degradation domain^40^. The N-terminal transactivation domain has variations that influence target gene specificity^41^. Although the interplay between HIF-1α and HIF-2α plays a crucial role in the context of obesity^42^, HIF-1α deletion has a protective effect on obesity, resulting in decreased insulin tolerance and adipogenesis^29,42^. Conversely, the absence or deficiency of HIF-2α leads to increased body weight, diminished glucose tolerance and insulin sensitivity in HFD-fed mice^42^. HIF-2α may suppress the activity and expression of HIF-1α. HIF-1α potentially plays a prominent role in LD formation and the storage of lipids and fatty acids in the small intestine, particularly during prolonged HFD consumption or obesity.

VHL is an enzyme that recognizes and binds to the hydroxyl group of HIF family members, inducing ubiquitination and proteasomal degradation under normoxic conditions^43^. Our results indicated that Golgi condensation occurs upon VHL depletion, regardless of CoCl_2_ treatment, as hydroxylated HIFs are not ubiquitinated or degraded in the absence of VHL. VHL depletion leads to the preservation of both HIF-1α and HIF-2α. Furthermore, our findings indicate that Golgi condensation is not prevented by depleting HIF-2α, in contrast to the effect observed with HIF-1α depletion. We also investigated HIF-1α target genes responsible for promoting Golgi condensation using siRNAs targeting several genes, including *Bnip3*, *Vegfa*, *Ca9*, *ADM* and *TPI1* (Supplementary Fig. 1c, d). Unfortunately, we were not able to define the exact gene that is regulated by HIF-1α and induces Golgi condensation. Further research is needed to elucidate the molecular mechanisms underlying this phenomenon. These results show that HIF-1α, rather than HIF-2α, plays a crucial role in enhancing Golgi condensation under hypoxic conditions.

The term ‘Golgi condensation’ was coined by Seith J et al.^30^, who reported that GOLPH3 depletion enhanced the shrunken structure of the Golgi apparatus by reducing the number of contact sites with actin. We elucidated the structure of the Golgi apparatus under hypoxic conditions. All hypoxic conditions, including the HFD, 1% O_2_, DMOG and CoCl_2_, could induce Golgi condensation with a reduction in GM130 expression. GM130 is a structural protein that is important for stacking *cis*-Golgi cisternae^44^. GOLPH3 binds to both Ptdlns(4)P and actin to maintain the linear structure of the Golgi apparatus^25^. The exact mechanism by which the depletion of either GM130 or GOLPH3 promotes Golgi condensation may differ. Similar to GOLPH3, GM130 depletion promoted the condensation of the Golgi apparatus due to HIF-1α activation, which impacts Golgi apparatus functions, such as fucosylation^10^.

Golgi proteins are degraded under certain conditions, including disturbances in Golgi homeostasis and osmotic stress^45^. For example, GM130 is degraded by the proteasome in the presence of lithocholylglycine, which enhances Golgi stress by inhibiting 2,3-linked sialyltransferase activity^5^. Recently, Golgiphagy was identified as a Golgi degradation pathway mediated by autophagy^46^. Although we documented the mechanism of Golgi condensation, the exact mechanism of Golgi condensation or Golgi protein degradation under hypoxic conditions is still not clear. GM130 mRNA expression was not decreased by hypoxia, whereas the GM130 protein level was notably decreased by proteasomal degradation. GM130 ubiquitination was also increased under hypoxic conditions, which was abolished by HIF-1α depletion.

NEDD4 has a WW domain for substrate binding and a HECT domain for E3 ligase activity. Typically, the substrate of NEDD4 contains a P(L)PxY domain that is recognized by the WW domain^47^. Our results showed that the expression and activation of NEDD4, which is regulated by HIF-1α, were elevated under hypoxic conditions. Moreover, GM130 degradation was prevented by NEDD4 depletion, which also reduced Golgi condensation under hypoxic conditions. However, GM130, which was ubiquitinated and decreased upon NEDD4 depletion, does not contain a P(L)PxY domain. Liu et al. reported that PTEN, which does not contain a PPxY domain, requires the adaptor protein Numb to bind NEDD4-1^48^. We speculate that an unknown adaptor protein with a P(L)PxY domain might bind to GM130 and support its interaction with NEDD4 for ubiquitination. NEDD4 suppression ameliorates metabolic disease phenotypes, such as increased lipolytic activity, decreased body weight and improved insulin tolerance, in HFD-fed mice^49^.

Hypoxia is an environmental condition that promotes lipid accumulation and increases inflammation in several different tissues^29^. The Golgi structural protein GRASP55 is involved in lipid accumulation in epithelial cells when the lipid trafficking pathway is interrupted^2^. Among the several pathways inducing lipid accumulation, we focused on how structural modifications of the Golgi apparatus impair lipid trafficking and promote lipid accumulation in the small intestine. Golgi condensation mediated by *Golph3* depletion increased the BODIPY and ApoA1 intensities but reduced the GM130 intensity. This evidence demonstrates that structural changes in the Golgi apparatus always reflect functional deterioration of the Golgi apparatus, including lipid trafficking and lipoprotein secretion, in the small intestine. We also evaluated the potential roles of other hypoxia-inducible factors, including DGAT-1, I-FABP and ACOX-1. However, these proteins were not involved in our experimental models.

Chylomicrons and HDL are lipoproteins that transport triglycerides and cholesterol to other tissues^50^. ApoA1 overexpression ameliorates obesity phenotypes, such as lipid accumulation in hepatocytes^51^ and cholesterol accumulation in macrophages^52^. When lipoprotein production or maturation is impaired, excess LDs are observed in the small intestine^2^. The cause of lipid accumulation under hypoxic conditions may be the inability to secrete free fatty acids or cholesterol as lipoproteins such as chylomicrons and HDL, resulting in excessive lipid accumulation in the ER. ApoA1 retention in the ER is probably increased by reduced trafficking from the ER to the Golgi apparatus due to Golgi condensation under hypoxic conditions. We observed ApoA1 retention in epithelial cells of the small intestine in vivo and in vitro under hypoxic conditions. Consistent with these findings, ApoA1 retention was reduced by Golph3 depletion, suggesting that structural aspects of the Golgi apparatus are closely related to its function, including intracellular lipid trafficking and lipid accumulation. Furthermore, the inhibition of HIF-1α has the potential to ameliorate obesity phenotypes, such as increased insulin tolerance and adipogenesis^29^. PX-478 treatment also increased the serum HDL concentration in HFD-fed mice, prevented Golgi condensation, and improved lipid trafficking and HDL secretion.

PX-478 effectively prevents adipocyte expansion and protects against LPS-induced acute liver failure^53,54^. Although the effects of PX-478 have been investigated in many other models, few studies have been conducted in intestinal models. For example, Morris et al. reported that PX-478 reduces HIF-1α expression caused by ethanol or burn injury in epithelial cells^55^, indicating an improvement in gut barrier quality. Another example is that PX-478 also decreases HIF-1α expression activated by exercise^56^. Similarly, our data demonstrate that PX-478 prevents HFD-induced HIF-1α expression (Fig. 8b). Collectively, these studies suggest that PX-478 effectively reduces HIF-1α expression in epithelial cells.

In this study, we focus on the morphology of the Golgi apparatus and lipid accumulation. Therefore, we provide several insights related to lipid secretion in epithelial cells under hypoxic conditions. However, information on lipid absorption under hypoxic or HFD conditions is limited. Kim et al. report that a deficiency of the Golgi structural protein GRASP55 reduces lipid absorption in intestinal epithelial cells^2^. This finding contradicts the findings of our study, as we observed that lipids were accumulated via Golgi condensation. Two possible explanations for the discrepancy between the two studies exist. First, we adapted cells and animals to either hypoxia or HIF-1α modulation throughout the experiments. Several studies have reported that HIF-1α increases free fatty acid absorption through PPARγ activation or the induction of FABP expression^57,58^. HIF-1α activation might promote lipid absorption independent of Golgi condensation. We showed that fatty acid uptake was increased following CoCl_2_ treatment (Supplementary Fig. 4c). However, this induction was reduced by PX-478 in the presence of CoCl_2_ (Supplementary Fig. 4c). The second possibility is due to the unique features of GRASP55. Kim et al. demonstrated that GRASP55 binds to ATGL, which is a crucial enzyme for lipolysis. Since ATGL cannot be localized due to GRASP55 deficiency, it might affect lipid absorption in intestinal epithelial cells^2^. Additional studies are needed to determine whether these findings are related to GM130 deficiency.

Taken together, our findings suggest that HIF-1α is a critical transcription factor that induces GM130 degradation through ubiquitination for Golgi condensation, resulting in lipid accumulation in vivo and in vitro. Our data also indicate that NEDD4 is an E3 ligase required for GM130 ubiquitination under hypoxic conditions. These results suggest that the prevention of Golgi condensation by the inhibition of HIF-1α activation has a therapeutic effect on the fatty intestine via the promotion of lipid trafficking, which results in lipid accumulation in the small intestine.

## Supplementary information

Supplementary Information
