# Supplementary material for: Golgi condensation causes intestinal lipid accumulation through HIF-1α-mediated GM130 ubiquitination by NEDD4
Source: Exp Mol Med. 2025 Feb 3;57(2):349–63. doi: 10.1038/s12276-025-01396-2 (PMC11873256; doi:10.1038/s12276-025-01396-2)
Supplement: Supplementary file 1 — Supplementary Information [file 12276_2025_1396_MOESM1_ESM.pdf]

Supplementary information

Golgi condensation causes intestinal lipid accumulation through HIF-1α-mediated GM130 ubiquitination by NEDD4

Hyunsoo Kim[1], Channy Park[1], Xiaofan Wei[1], Arun Chhetri[1], Laxman Manandhar[1], Gyuho Jang[1], Jaetaek Hwang[1], Batchingis Chinbold[1], Chagtsalmaa Chuluunbaatar[1], Hyug Moo Kwon[2], and Raekil Park[1*]

[1]Department of Biomedical Science and Engineering, Gwangju Institute of Science and Technology, Gwangju 61005, Republic of Korea

[2]School of Life Sciences, Ulsan National Institute of Science and Technology, Ulsan, Republic of Korea

* Correspondence to:

Raekil Park, M.D, Ph.D.

Department of Biomedical Science & Engineering,

Gwangju Institute of Science and Technology, Gwangju 61005, Republic of Korea

Tel.: +82-62-715-5361; Fax: +82-62-715-5309; E-mail: rkpark@gist.ac.kr

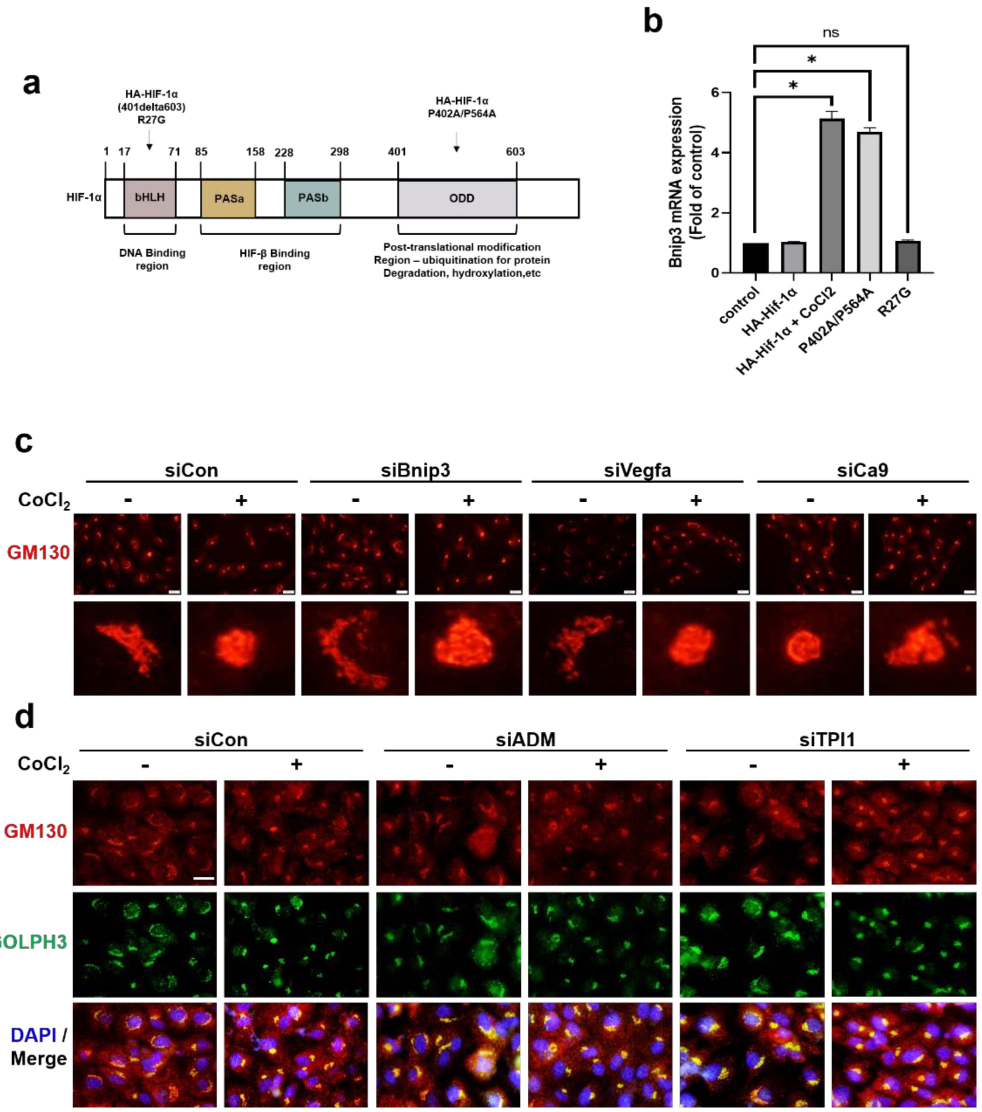

Supplementary Fig. 1. HIF-1α activation is required for Golgi condensation, which is not affected by HIF-1α target genes upon $CoCl_2$ in RPE1 cells.

a. Schematic map for HIF-1α mutation loci. **b**. mRNA expression of *Bnip3* was quantified by qRT-PCR in the indicated experimental conditions. Data represent the mean ± S.D (n = 3, independent experiments). *$p < 0.05$. **c**. Protein expression of GM130 was observed by immunofluorescence in various depletion conditions of HIF-1α target gene. Scale bar, 5 μm. **d**. Protein expression of GM130 and GOLPH3 was tested by immunofluorescence in various depletion conditions of HIF-1α target gene. Scale bar, 20 μm.

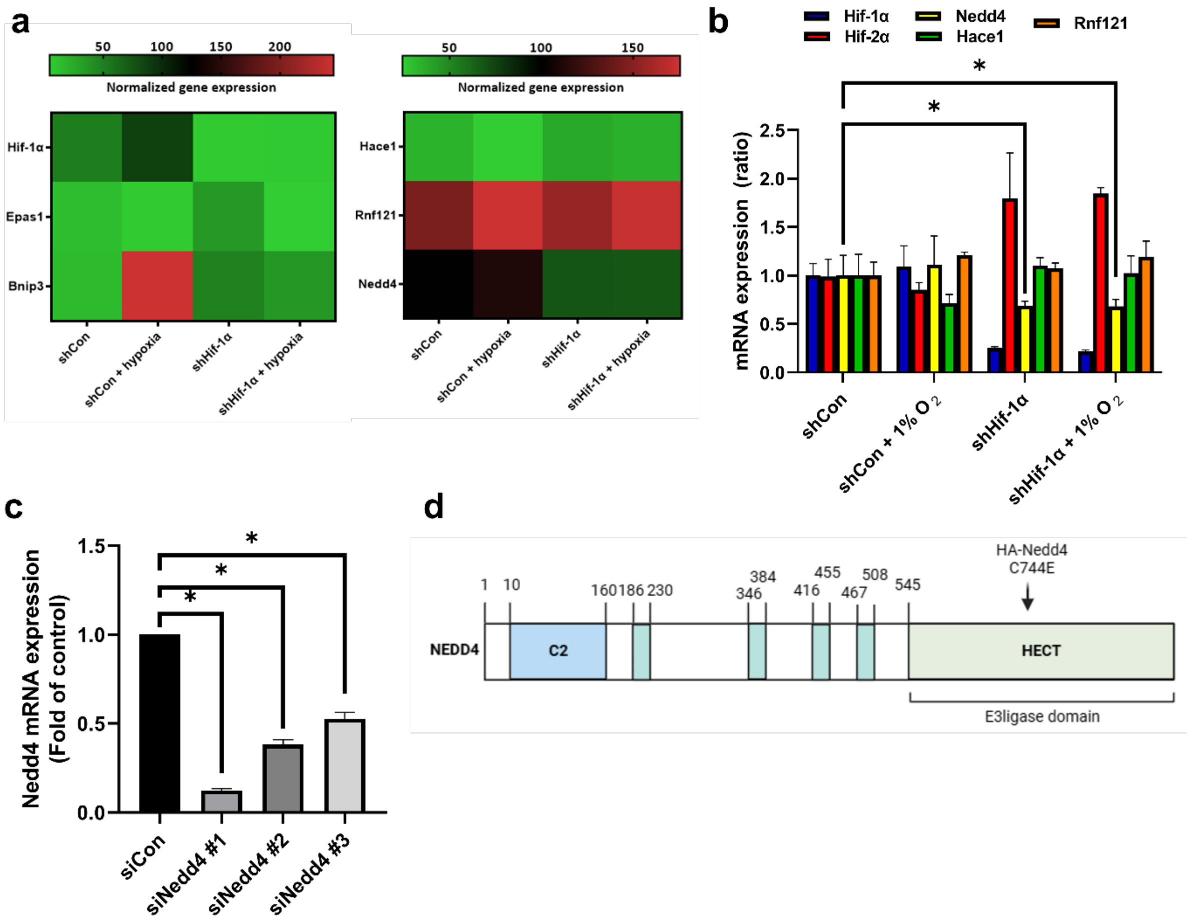

Supplementary Fig. 2. Nedd4 is a potential candidate of E3 ligase involving in hypoxia.

a. Heat map. **b**. RPE1 cells were incubated in 1% O_2 and mRNA expression was quantified by qRT-PCR. Data represent the mean ± S.D (n = 3 independent experiments), *p < 0.05. **c**. Transfection efficiency of siNEDD4 was evaluated by qRT-PCR. Data represent the mean ± S.D (n = 3, independent experiments), *p < 0.05. **d**. Schematic map of NEDD4 mutation loci.

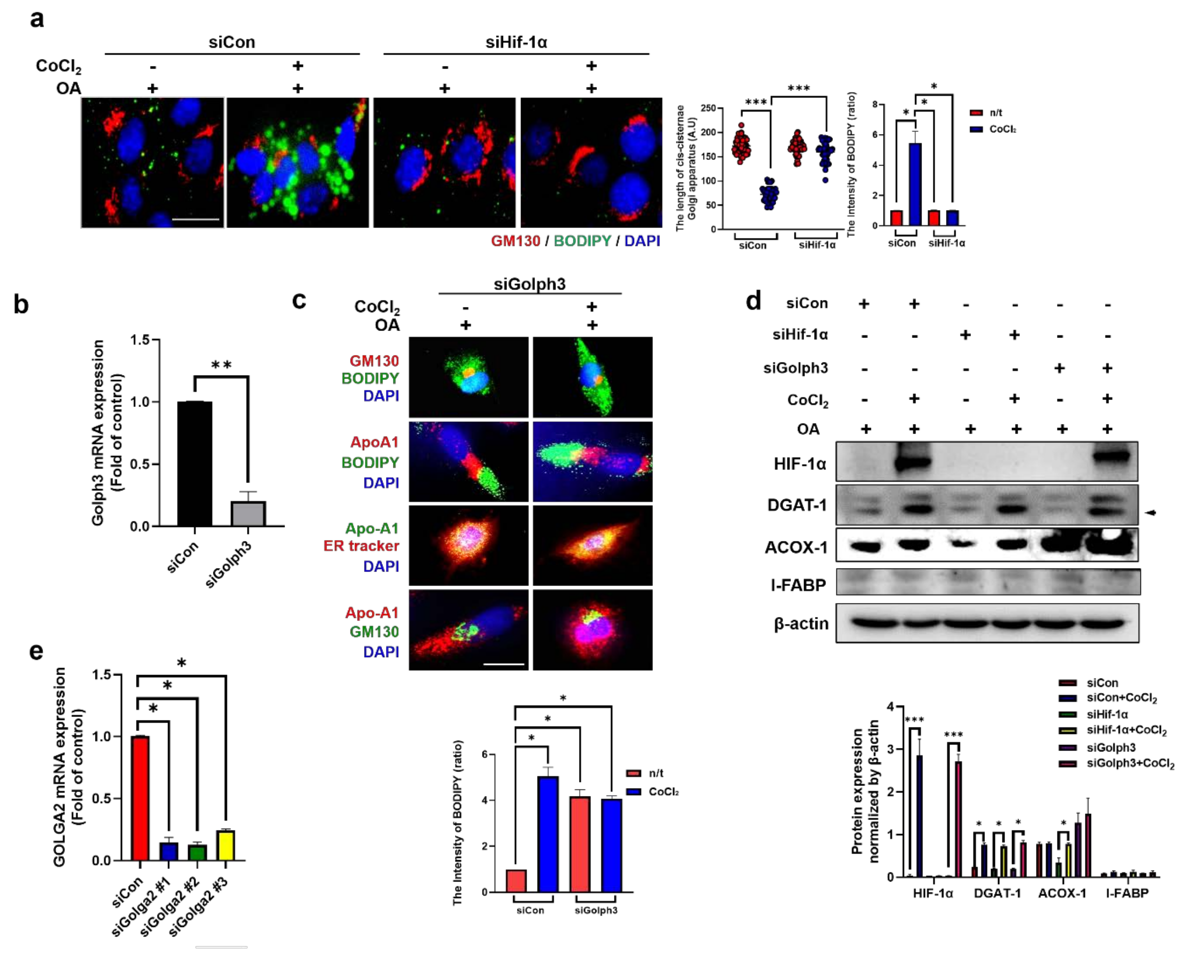

Supplementary Fig. 3. Golph3 depletion induces Golgi condensation and does not affect the lipid-related pathways irrespective of $CoCl_2$ treatment.

a. Caco-2 cells transfected with siCon or siHIF-1α were incubated in oleic acid (OA) contained medium in the absence or presence of $CoCl_2$ for 24 h. Cells were subjected to immunofluorescence analysis for GM130 and BODIPY to visualize Golgi and lipids. Statistical histogram of Golgi cisternae length and BODIPY intensity. Scale bar, 10 µm. Data represented the mean ± S.D (n = 5 independent experiments), *p < 0.05, and ***p<0.001. (**b-d**). Fhs74int cells transfected with siGolph3 were incubated in oleic acid (OA) contained medium in the absence or presence of $CoCl_2$ for 24 h. **b**. mRNA expression of *Golph3* was measured by qRT-PCR after siGolph3 transfection. Data represent the mean ± S.D (n = 5, independent experiments), **p < 0.01. **c**. Cells were subjected to immunofluorescence analysis for GM130 and BODIPY to visualize Golgi and lipids; Apo-A1 for lipoproteins (chylomicron/HDL), ER tracker for ER, and DAPI for the nuclei, respectively. Scale bar, 5 µm. The intensity of BODIPY was measured in graph. Data represent the mean ± S.D (n = 5 independent experiments), *p < 0.05. **d**.

Protein expression of HIF-1α, DGAT-1, ACOX1, I-FBA, and β-actin was analyzed by Western blot. Protein expression normalized by β-actin was measured with statistical analysis. Data represented the mean ± S.D (n = 3 independent experiments), *$p<0.05$, ***$p<0.001$. **e.** mRNA expression of Golga2 was measured by qRT-PCR after siGolga2 transfection. Data represent the mean ± S.D (n = 5, independent experiments), **$p < 0.01$.

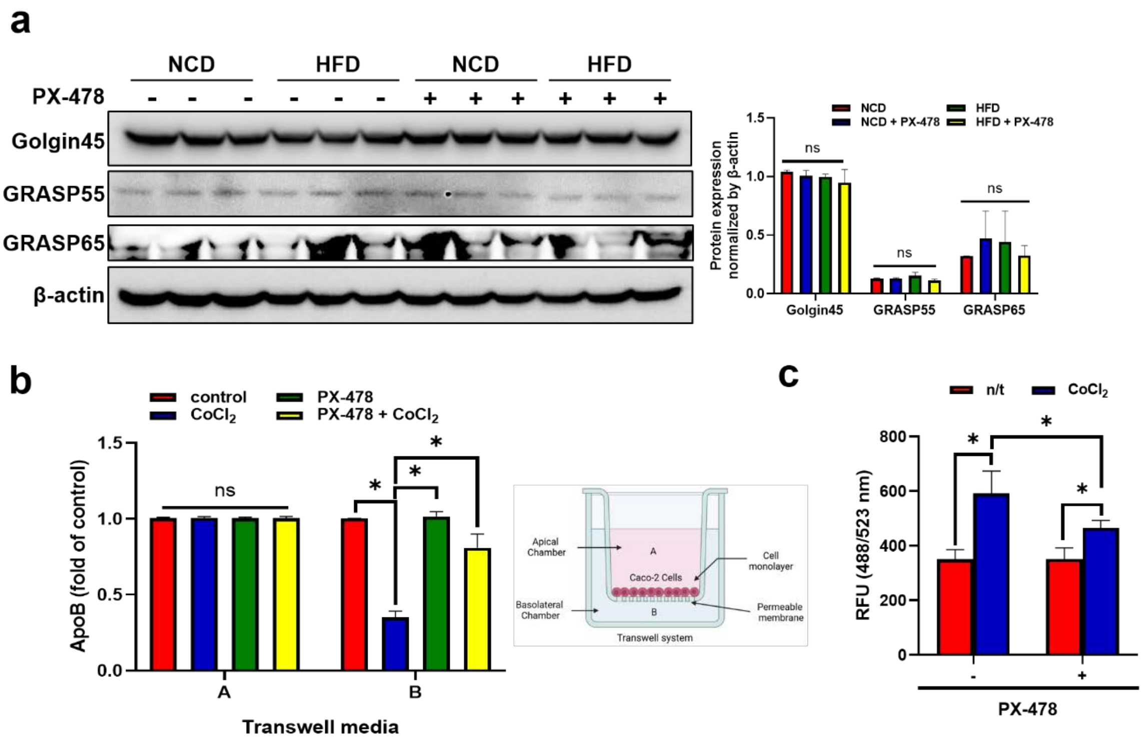

Supplementary Fig. 4. Expression of Golgi structural proteins is not reduced by HFD, however reduced chylomicron secretion induced by $CoCl_2$ is rescued by PX-478 treatment.

a. Eight-week old mice were fed with either NCD or HFD for 16 weeks. PX-478 (10 mg/kg) was administered via oral gavage thrice a week starting at 6 weeks of HFD feeding. Protein expression of Golgin45, GRASP55, GRASP65, and β-actin was measured by immunoblotting. Protein expression normalized by β-actin was measured with statistical analysis. Data represented the mean ± S.D (n = 5 independent experiments), *p<0.05. **b-c.** Caco-2 cells were treated with PX-478 prior to $CoCl_2$ treatment. **b**. ApoB secretion was measured using transwell system. Media from Apical area (A) and Basolateral area (B) was collected to determine the ApoB concentration which was measured by ELISA. Data represented the mean ± S.D (n = 5 independent experiments), *p<0.05. **c.** Free fatty acids uptake amounts were determined by RFU (488/523 nm). Data represented the mean ± S.D (n = 5 independent experiments), *p<0.05.

Supplementary Fig. 5. Tables

a. Table 1. siRNA sequences

Target	Direction	Sequence
Hif-1α	F	GUG GUU GGA UCU AAC ACU
	R	UAG UGU UAG AUC CAA CCA
Epas1 (Hif-2α)	F	CGU GAG AAC CUG AGU CUC A
	R	UGA GAC UCA GGU UCU CAC
pVHL	F	ACU GAU GAG UCU UGA UCU A
	R	UAG AUC AAG ACU CAU CAG U
Nedd4	F	GAG UUG CCA GAG AAU GGU U
	R	AAC CAU UCU CUG GCA ACU C
Golga2	R	AAC GAC CGC ACT ACC ATC
	F	GGT GAT CTC CAT GTT CTC ATT AG

b. Table 2. Primer sequences

Target	Direction	Sequence
Hif-1α (h)	F	CGT TGT GAG TGG TAT TAT TCA GC
	R	GAG GCT ACT TGT ATC TTC TGA TTC AAC
Hif-1α (m)	F	TGC TCA TCA GTT GCC ACT TC
	R	TGG GCC ATT TCT GTG TGT AA
Ca-9	F	GCT GAA CTT CCG AGC GA
	R	GAT GTC ACC AGC AGC CAG
Bnip3 (h)	F	GAA TTT CTG AAA GTT TTC CTT CCA
	R	TTG TCA GAC GCC TTC CAA TA
Bnip3 (m)	F	GCT CCC AGA CAC CAC AAG AT
	R	TGA GAG TAG CTG TGC GCT TC
Bnip3 (Chip)	F	CCT AGC TAG CCG GTC CAC TTC TGC ATT AGA CC
	R	CGG AGG ATC TTG CGC CGC TCA GTT CTG AGG CCA GG
Nedd4	F	TTG CAC TTT GCA GCC AGA AG
	R	CTG TCT GGG AGT CAG CTT CA
Golph3	F	CUC AAG UAA ACC AGU AGU U
	R	AAC UAC UGG UUU ACU UGA G
Golga2	F	GAG AGU ACA UUG CAC UGU A
	R	UAC AGU GCA AUG UAC UCU C

c. Table 3. Antibodies

Proteins	Origin	Antibody details
β-actin	Mouse	Santa Cruz Biotechnology (sc-47778)
HIF-1α	Rabbit	Abcam (ab51608)
HIF-1α	Mouse	BD transduction Laboratories (610958)
GM130	Mouse	BD transduction Laboratories (610822)
GOLPH3	Rabbit	SIGMA (SAB4200341)
PLIN2	Rabbit	Abcam (ab108323)

HA	Rabbit	Sigma (H6908)
CREB	Rabbit	Cell Signialling (4820S)
α-tubulin	Mouse	Santa Cruz Biotechnology (sc-58666)
GALNT2	Rabbit	Proteintech (17441-1-AP)
Golgin45	Rabbit	Proteintech (16354-1-AP)
GRASP65	Rabbit	Abcam (ab174834)
GRASP55	Rabbit	Abcam (ab204335)
Alexa Flour 488 goat anti-rabbit IgG (H+L)	Goat	Invitrogen (A11034)
Alexa Flour 568 goat anti-mouse IgG (H+L)	Goat	Invitrogen (A11031)
Goat-x-Rabbit IgG-Fc-Fragment-HRP Conjugated	Goat	BETHYL (A120-111P)
Goat-x-Mouse IgG-Fc-Fragment-HRP Conjugated	Goat	BETYL (A90-131P)
